# Comparative analysis of the mobilome yields new insights into its diversity, dynamics and evolution in parasites of the Trypanosomatidae family

**DOI:** 10.1017/S0031182025100231

**Published:** 2025-05

**Authors:** Percy Omar Tullume-Vergara, Adriana Ludwig, Vyacheslav Yurchenko, Adriano Cappellazzo Coelho, Elizabeth Magiolo Coser, Marco Aurélio Krieger, Marta M.G. Teixeira, Jeffrey Jon Shaw, Joao M.P. Alves

**Affiliations:** 1 Department of Parasitology, Institute for Biomedical Sciences, University of Sao Paulo, Sao Paulo, Brazil; 2 Department of Evolution, Ecology and Behaviour, University of Liverpool, Liverpool, UK; 3 Life Science Research Centre, Faculty of Science, University of Ostrava, Ostrava, Czech Republic; 4 Departamento de Biologia Animal, Instituto de Biologia, Universidade Estadual de Campinas (UNICAMP), Campinas, Brazil; 5 Vice Presidency of Production and Innovation in Health (VPPIS), Oswaldo Cruz Foundation (FIOCRUZ), Rio de Janeiro, Brazil

**Keywords:** CRE, INGI, mobilome, SLACS, TATE, transposable elements, trypanosomatids, VIPER

## Abstract

Transposable elements (TEs) have the ability to move and amplify inside the host genome, making them a pivotal source of genome plasticity. Presently, only 4 TE clades (all classified as Class I retrotransposons) have been identified in trypanosomatids. We predicted repeat content and manually curated TEs across the genomes of 57 trypanosomatids, shedding light on their proportions, diversity and dynamics. Our analysis yielded 214 TE consensus sequence models across the dataset, with abundance ranging from 0.1% to 7.2%. We found evidence of recent transposon activity in most species, with notable bursts in the *Vickermania, Lafontella, Porcisia* and *Angomonas* spp., along with *Leishmania (Mundinia) chancei, L. (M.) orientalis* and *L. (M.) procaviensis*. We confirmed that the 4 TE clades have colonized virtually all lineages of trypanosomatids, potentially playing a role in shaping their genome architecture. The effort of this work culminated in the establishment of the Trypanosomatid TE Database 1.0, a resource designed to standardize the TE annotation process that can serve as a foundation for future studies on trypanosomatid TEs.

## Graphical abstract

**Figure:**
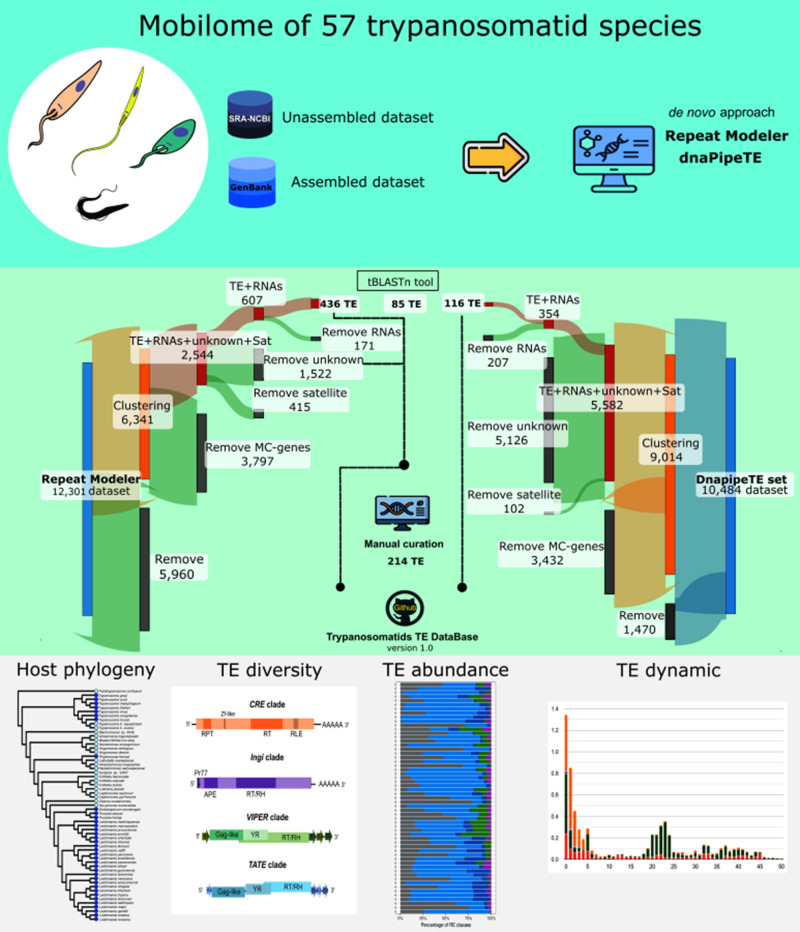

## Introduction

Eukaryotic genomes harbour a significant fraction of repetitive elements (REs), which play a determinant role in driving genetic innovation in the genome (Kazazian, 2004; Bourque et al., 2018). Significant repeat categories that comprise the ‘repeatome’ include transposable elements (TEs) and certain protein-coding gene families, which are dispersed throughout the genome. Another major category consists of tandem repeats, such as satellite DNA, ribosomal RNA and simple repeats, which are arranged in consecutive copies along the genomic DNA (Woo et al., 2007; Biscotti et al., 2015). In certain organisms, TEs are the predominant type of RE within the eukaryotic genome. They were first discovered by Barbara McClintock, who referred to them as ‘controlling elements’ (McClintock, 1984). It is well known that TEs are constituted by a large variety of families that can be categorized into 2 major classes based on their transposition mechanisms: Class I TEs (retrotransposons), which relocate into the genome through a ‘copy-and-paste’ mechanism involving an RNA intermediate, and Class II TEs (DNA transposons), which move *via* a DNA intermediate mostly using ‘cut-and-paste’ mechanism of mobilization (Finnegan, 1989; Bourque et al., 2018; Gilbert et al., 2021). The retrotransposons are divided into 5 orders distinguished by the major organizational structures of their coding and non-coding domains: LTR (long terminal repeat) retrotransposons, LINE (long interspersed nuclear elements), DIRS (*Dictyostelium* intermediate repeat sequence) elements, PLE (*Penelope*-like elements) and SINE (short interspersed nuclear elements) (Wicker et al., 2007).

The family Trypanosomatidae contains a number of parasitic protistan lineages that can be divided into 2 major non-taxonomic groups: monoxenous (only 1 host, parasitizing mostly invertebrates) and dixenous (alternating between invertebrates, vertebrates and, sometimes, plants) organisms (Kaufer et al., 2017; Kostygov et al., 2021). Earlier studies have evaluated the repetitive content of the main medically important trypanosomatid species (*Trypanosoma brucei, T. cruzi* and *Leishmania major*) and reported retrotransposons (2–5%) as the only colonizers, while not documentingthe presence of DNA transposons (Bringaud et al., 2007; Macías et al., 2018). More recently, using graph-based clustering of short reads, a proportion of TEs in the *T. brucei* and *T. cruzi* genomes was estimated to be even higher, at ∼6% and 13%, respectively (Pita et al., 2019).

**Figure 1.**
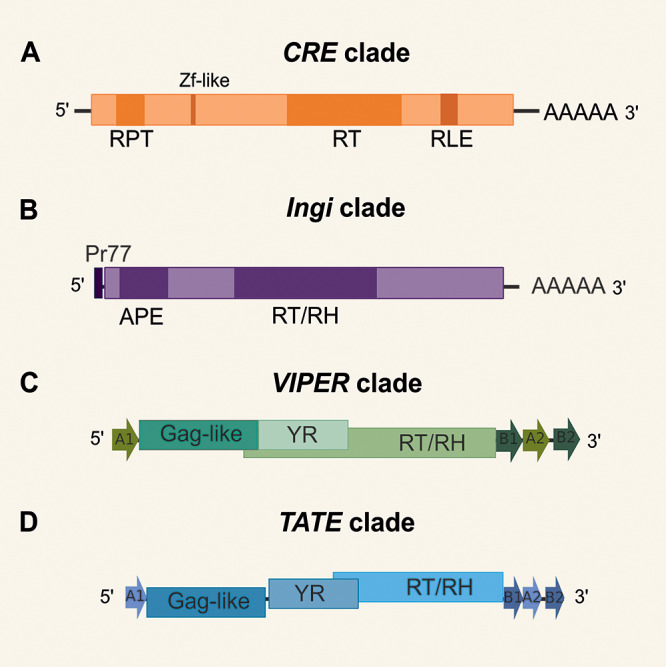
Schematic structure of autonomous elements from each known clade of TE in trypanosomatids. ORFs are shown as long rectangles, and the size of elements varies within clades (not drawn to scale). (A) *CRE* clade, containing: reverse transcriptase (RT), restriction enzyme-like endonuclease motif (RLE), a poly-A tail, 1 or 2 internal repeat regions (RPT); most copies present 1 or 2 zinc finger-like structures. (B) Autonomous *INGI* elements, containing: apurinic/apyrimidinic endonuclease (APE), RT and RNase h (RH), a highly conserved 77-nt sequence (pr77), a variable-length poly(A) tail. (C) *VIPER* (vestigial interposed retroelement) and (D) *TATE* (telomerase-associated transposable element) clades have similar structures, containing: a putative gag-like gene, 2 overlapped ORFs encoding for tyrosine recombinase (YR) and RT/RH, split direct repeats (SDRs), represented as arrows A1 at the 5′ end and B1, A2 and B2 at the 3′ end.

Concerning TE diversity in trypanosomatids, older studies have reported only 4 major clades, namely *INGI, CRE, VIPER* and *TATE* (Aksoy, 1991; Vazquez et al., 2000; Bringaud et al., 2002; Lorenzi et al., 2006; Peacock et al., 2007) (Figure 1). The *INGI* and *CRE* clades belong to the LINE order (also known as non-LTR retrotransposons) (Kojima, 2020). The *CRE* clade (Figure 1A) comprises a group of elements originally identified by different names (SLACS, CZAR, CRE1 and CRE2) which consistently insert at the same relative position in the spliced leader (SL) RNA genes (Aksoy, 1991; Teng et al., 1995). While these elements were previously thought to encode 2 open reading frames (ORFs) in *T. cruzi*, we verified a single ORF in most species. These elements encode a reverse transcriptase (RT) and possess a restriction enzyme-like endonuclease motif (RLE). In general, the 3ʹ end is characterized by a poly-A tail. Most copies present 1 or 2 zinc finger (ZF)-like motifs (Fujiwara, 2015). Additionally, 1 or 2 internal repeat regions (RPT) are found in CRE elements of some species. These elements generate target-site duplications (TSDs, not represented in Figure 1) that vary in size.

Potentially complete *INGI* elements were found in *T. brucei* (Tbingi), *T. congolense* (Tcoingi), *T. cruzi* (L1Tc) and *T. vivax* (Tvingi) (Bringaud et al., 2009), while only remnants of the complete *INGI* were detected in *L. major* (DIREs) (Bringaud et al., 2008). Autonomous INGI elements (Figure 1B) encode an apurinic/apyrimidinic endonuclease (APE), RT and RNase H (RH). In the 5ʹ end, these elements possess a highly conserved 77-nt sequence (Pr77) that works as a DNA promoter (Heras et al., 2007) and has a ribozyme activity (Sánchez-Luque et al., 2011). They have a variable-length poly(A) tail and generate TSDs (not represented in the figure). Short non-autonomous versions of *INGI* known as TbRIME, NARTc, LmSIDER and LbSIDER (not represented in Figure 1) are found in some species (Bringaud et al., 2002; Smith et al., 2009).

While there were no reports of typical LTR elements in trypanosomatid genomes, *VIPER* (vestigial interposed retroelement) and *TATE* (telomerase-associated transposable element) elements of the DIRS order (Wicker et al., 2007) were included in the LTR group in the Repbase classification (Finnegan, 1989; Kojima, 2020). *VIPER* retrotransposon was initially described as a degenerated TE family in *T. cruzi* (Vazquez et al., 2000) and more recently found in several trypanosomatid genomes, being potentially active in some species, including *T. cruzi* itself (Ribeiro et al., 2019). Besides, the *TATE* superfamily was initially discovered in *Leishmania* spp. of the subgenus *Viannia* (Peacock et al., 2007; Llanes et al., 2015) and later degenerate and potentially active copies of *TATE* were found in other trypanosomatid genomes (Ribeiro et al., 2019). Autonomous *VIPER* and *TATE* elements have a similar structure (Figure 1C and D). They encode a first ORF considered a putative Gag-like gene and 2 additional overlapped ORFs encoding for tyrosine recombinase (YR) and RT/RH. Both clades have split direct repeats (SDRs) represented as arrows A1 at the 5′ end and B1, A2 and B2 at the 3′ end. *VIPER* and *TATE* elements do not generate TSDs upon insertion. A short version of *VIPER*, called SIRE (short interspersed repetitive element) was identified in *T. cruzi* (Vazquez et al., 2000) and corresponds to the region encompassing A2 and B2 repeats (Ribeiro et al., 2019).

Currently, when genome assemblies are published, their repetitive content is often treated superficially, without any curation step, or overlooked altogether, which results in incomplete and, sometimes, inaccurate characterization of repeat elements, specifically TEs (Goubert et al., 2022). There is little doubt that an in-depth analysis of the evolutionary history of TEs across a broad range of non-model trypanosomatid species is essential to elucidate the diversity and dynamics of these sequences within this group. The current study delves into the mobilome of 57 genomes of the members of the family Trypanosomatidae and explores the abundance, superfamily composition and evolutionary dynamics of TEs. In addition, we report the establishment of a custom expanded TE library for genome annotation of new trypanosomatid sequences that will provide a valuable resource for future studies on TEs.

## Materials and methods

### Acquisition, quality check and preprocessing of the genomic dataset

In this study, we have compiled 2 datasets comprising assembled and unassembled genomes utilized for the analysis of TEs across 57 species from the family Trypanosomatidae (Albanaz et al., 2023; Kostygov et al., 2024). The first dataset was constituted by genome assemblies, retrieved from the National Center for Biotechnology Information (NCBI) GenBank (Sayers et al., 2021) [accessed 07/15/2024] and from the TriTryp database release 58.0 (Shanmugasundram et al., 2023). We assessed gene completeness by employing the ‘Benchmarking Universal Single Copy Orthologs’ (BUSCO) tool v. 5.4.3 (Seppey et al., 2019) using mode: -genome, against the Euglenozoa database (Euglenozoa_db10), comprising 130 BUSCO orthologous genes (Kuznetsov et al., 2023). Furthermore, to estimate the contiguity of the genomes, we calculated the contig N_50_ values (Supplementary Figure S1). To build RE models with RepeatModeler v. 1.0.8 (Flynn et al., 2020), we included only high-quality assemblies (37 genomes in total), selecting only 1 genome per species based on the longest contig N_50_ value (Supplementary Table S1).

A second dataset comprising short-read genomic libraries from 20 trypanosomatid species was downloaded from the NCBI Sequence Read Archive (Katz et al., 2022). The short reads were trimmed with fastp v. 0.19.4 (Chen et al., 2018) using the following settings: --length-limit 50 -f 5 -t 5 -F 5 -T 5 in order to eliminate low-quality sequences and read quality was checked using fastQC v. 0.11.8 (Andrews, 2010) before and after the trimming step. We estimated the haploid genome size for the unassembled short-read genomes by applying *k*-mer frequency counting using Jellyfish v. 2.2.10 (Marçais and Kingsford, 2011) and this estimate was further used to infer the repetitive content fraction. The genome size distribution profiles were visualized using the Genoscope2 web tool (Ranallo-Benavidez et al., 2020) (Supplementary Figure S2). A list of all genomes used in this study, including the source, name of isolate, contig N_50_ value, genome coverage, assembler and publication references is shown in Supplementary Table S1.

### Identifying RE content from genome assemblies, and curation and classification of TEs

Genome assemblies were used to build a *de novo* repeat library for each of the 37 species using RepeatModeler v. 1.0.8 (Flynn et al., 2020). To enhance the probability of finding new TEs, we additionally employed tools with a structure-based component: LTR_retriever v. 2.9.0 (Ou and Jiang, 2018), which uses accurate REs input from the LTR_Finder v. 1.07 (Xu and Wang, 2007), and LTR_harvest v. 1.5.10 (Ellinghaus et al., 2008), to predict complete LTR retrotransposons.

For each resulting RE library, filtering steps were applied to remove spurious candidate models not associated with TEs (i.e., multicopy genes or tandem repeats). Tandem repeats were identified with Tandem Repeats Finder v. 4.09 (Benson, 1999) with a threshold of 30%. Additionally, we used tRNAScan-SE v. 2.0.3 and cmscan (Chan et al., 2021) on each raw library to identify and quantify tRNA, rRNAs and snoRNAs, and the Blastx tool against Uniref-90 (UniProt Reference Clusters) (Suzek et al., 2015) and non-redundant NCBI databases [assessed 12/20/2023] to identify putative TEs and protein families (GP63, amastin, sialidases, GP46, tubulin, heat shock protein, etc.) within the raw libraries.

Sequences classified as unknown that were not eliminated in the previous filtering steps or confirmed as having some TE-related domain(s) were further screened using the web version of Censor (Kohany et al., 2006) to analyse potential similarities to known TEs, including non-autonomous ones. TE-aid (Goubert et al., 2022) was used to visualize the structure (potential ORFs and terminal repeats) and genomic coverage of the consensuses. Online Conserved Domain search (Wang et al., 2023) was employed to search for protein domains in sequences with a higher potential to be TEs, such as those with a defined structure or of a larger size. Only sequences with TE domains or TE-similarity were maintained in the consensus sequence curation steps.

Next, we performed a manual curation of all the potential TEs (consensus sequences) within each library generated by RepeatModeler following the main steps outlined previously (Goubert et al., 2022). Briefly (i) to remove redundant consensus sequences, we ran CD-HIT-EST v. 4.8.1 (Weizhong and Godzik, 2006) with the following settings: -c 0.80 -n 5 -M 0 -aS 0.80 -g 1 -G 0 over the filtered REs library expecting to meet the majority 80-80-80 rule for the classification of the TE family (Wicker et al., 2007); (ii) next, the TE library was split into individual consensus sequences using split-fasta v. 3.6 (https://pypi.org/project/split-fasta/); (iii) to find all members of each family, the bash script ‘make_fasta_from_blast.sh’ (Goubert et al., 2022) was used, performing a Blastn v. 2.5.0 + (Camacho et al., 2009) search against the formatted genomes and retrieving extended sequences (±0.5–1.5 kbp) to include as much of the ends of the TEs as possible; (iv) all recovered sequences were aligned using MAFFT v. 7.453 (Katoh and Standley, 2013); (v) the resulting MAFFT alignments were manually inspected in Aliview.jar v. 2021 (Larsson, 2014) to delimit the elements and remove flanking sequences and indels; (vi) finally, the cons tool from the EMBOSS package v. 6.6.0.0 (Rice et al., 2000) was used to obtain the consensus sequence of autonomous and non-autonomous TEs. In the aforementioned steps, we also used Bedtools v. 2.27.1 (Aaron et al., 2010; Quinlan, 2014) or ‘make_fasta_from_blast.sh’ script, to extract TE copy from genome assembly and extend the length of their flanking regions. During our curation analysis, TE-trimmer, a tool to aid the manual curation of TE libraries (Qian et al., 2024), was published. We tested it and found it helpful in visualizing the PFAM protein domains (Mistry et al., 2021); however, we observed that the consensus sequences were frequently incorrect, particularly shrinking the *DIRS* and extending the *CRE* elements.

To help delimit some elements, we used Blastn with the parameters ‘align 2 or more sequences’ and ‘somewhat similar sequences (blastn),’ adjusting the *e*-value threshold to 10 and using the same sequence as the query and subject. This strategy was used to find the target site duplications (TSDs) generated by *CRE* elements since their consensus is often extended due to their insertion in a repetitive region. Moreover, this was also used to identify the SDRs of *VIPER* and *TATE*.

For the purpose of classification, the consensus sequences were categorized according to their similarity to known TEs in the Repbase Database (Bao et al., 2015) utilizing the web browser version of Censor (Kohany et al., 2006). The sequences were named sequentially using a format that includes the species name abbreviation, a unique identifier, and the TE family classification with the order and clade/superfamily information (*INGI, CRE, TATE* or *VIPER*) (e.g., Tcru-1#LINE/CRE). Some known TE families were not repetitive enough in some of the genomes to be identified by RepeatModeler. To overcome this, a Tblastn search was performed against each genome. The *TATE, VIPER, CRE* and *INGI* canonical proteins from Repbase Database were used as queries using an E-value threshold of 1e-9. The sequences were retrieved using getfasta from the Bedtools package, and the steps described above were followed to retrieve the copies and make a consensus sequence, whose classification was confirmed using the Censor webtool.

Using the approach specified above, 1 accurate species-specific TE library was obtained for each genome assembly. These custom TE libraries were merged for downstream analysis by dnaPipeTE (Goubert, 2023). All of these procedures resulted in the creation of the custom Trypanosomatid TE DataBase v. 1.0 available on GitHub (https://github.com/percytullume/TEs_trypanosomatids).

### Improving the annotation provided by RepeatMasker to infer copy number

To get the annotation of the TEs, each of the 37 curated species-specific TE libraries was mapped against its corresponding genome assembly using RepeatMasker v. 4.1.2 (Tarailo-Graovac and Chen, 2009) with the following options: -s -excln -a -gccal -norna -lib -nolow. To improve the accuracy of copy number estimation, we utilized the ‘One-code-to-find-them-all’ script (Bailly-Bechet et al., 2014), which provides precise TE copy coordinates and accurate quantification of TE families (Table 1). There was no inference of TE copy numbers for unassembled genomes since the dnaPipeTE tool uses a low coverage genome (∼0.15 ×); therefore, the retrieved elements are frequently fragmented, generating unreliable estimates of copy number.

**Table 1.** Diversity of Class I TEs in trypanosomatid genomes

Species	*INGI*	*CRE*	*TATE*	*VIPER*
*Angomonas deanei*	33	11	104	1
*Blechomonas nonstop*	11	5	182	0
*Crithidia bombi*	20	1	9	21
*Crithidia expoeki*	111	6	14	37
*Crithidia fasciculata*	49	10	299	157
*Herpetomonas samuelpessoai*	37	5	132	98
*Kentomonas sorsogonicus*	18	6	352	166
*Lafontella mariadeanei*	63	533	248	1185
*Leishmania aethiopica*	1671	0	8	0
*Leishmania amazonensis*	1077	0	3	0
*Leishmania braziliensis*	1308	11	69	37
*Leishmania chancei*	435	0	782	1
*Leishmania donovani*	1200	0	4	21
*Leishmania enriettii*	1118	0	445	2
*Leishmania guyanensis*	1422	7	102	118
*Leishmania infantum*	1293	0	4	43
*Leishmania lainsoni*	977	27	19	12
*Leishmania major*	1240	0	2	0
*Leishmania martiniquensis*	116	0	176	1
*Leishmania mexicana*	1437	0	7	0
*Leishmania orientalis*	996	0	751	2
*Leishmania procaviensis*	1002	0	465	3
*Leishmania shawi*	1345	6	48	106
*Leishmania tarentolae*	543	0	0	0
*Leishmania tropica*	1350	0	3	51
*Leptomonas pyrrhocoris*	2	14	64	250
*Lotmaria passim*	98	11	0	0
*Porcisia hertigi*	50	4	255	0
*Trypanosoma brucei*	1314	65	0	76
*Trypanosoma (b.) evansi*	295	6	0	56
*Trypanosoma (b.) equiperdum*	312	8	0	22
*Trypanosoma congolense*	451	7	0	188
*Trypanosoma cruzi*	439	775	0	1764*
*Trypanosoma melophagium*	148	3	22	167
*Trypanosoma vivax*	1189	78	0	805
*Vickermania ingenoplastis*	5	864	1237	863
*Zelonia costaricensis*	43	14	210	24

The number of copies in 4 superfamilies was assessed by RepeatMasker and the ‘one-code-to find-them-all’ script.

* The copy number of *VIPER* in *T. cruzi* also includes the short version, SIRE.

### Kimura distance-based distribution analysis

From the RepeatMasker output, the .tbl file was used to estimate the TE coverage (proportions) for all trypanosomatid genomes. In addition, the .align file was used to estimate the divergence of copies and their family consensus sequence using the Kimura distance model (Kimura 2-parameter [K2P]) (Kimura, 1980) with the calDivergeneceFromAlign.pl script from the RepeatMasker package. We employed the Kimura distances with correction for CpG pairs for all trypanosomatid genera except *Leishmania*, where the parameter -noCpGMod was used, since DNA methylation has been documented for *Trypanosoma* sp. but not *Leishmania* spp. (Militello et al., 2008; Cuypers et al., 2020). We also used the ‘createRepeatLandscape.pl’ script from RepeatMasker to generate landscape bar plots illustrating the temporal activity of TEs within the genomes. Furthermore, TE families were grouped into the orders LINE (*INGI* and *CRE*) and DIRS (*TATE* and *VIPER*).

### Repeat context analysis using the dnaPipeTE pipeline on the unassembled dataset

The second dataset included raw sequence read libraries from 20 trypanosomatid species. The abundance and proportion of each RE were estimated with the dnaPipeTE v. 1.4c (Goubert, 2023). The pipeline utilizes high-quality short-read sequencing libraries (either forward or reverse reads). Initially, to avoid overestimating REs, sequence reads aligning to the respective mitochondrial genomes were excluded for each species using the BBDuk package v. 37.62 from BBTools (Bushnell et al., 2017). dnaPipeTE uses Trinity v. 2.5.1 (Grabherr et al., 2011) to assemble RE contigs from low genome coverage (<1 × subsamples), enabling the identification of these sequences in species lacking high-quality genome assemblies. We performed tests of low coverage from 0.05 × to 0.21 × in intervals of 0.02 × (9 runs) on all datasets, as suggested by Goubert (2023) (summarized in Supplementary Table S2), to find the highest contig N_50_ (optimal assembly) in the assembly step of the pipeline (best coverage species-specific). To infer genome size for dnaPipeTE, we employed Genoscope2 (Supplementary Table S4; Supplementary Figure S2). To improve the classification accuracy and annotation of TEs, we ran dnaPipeTE twice: (i) in the first run, we used the trypanosomatid RepeatModeler library (obtained in this work as described above), with the following parameter: -RM_lib (custom library). This custom RepeatModeler library gathered 37 curated TE libraries to identify potential candidate TEs; (ii) in the second run, we employed the correctly classified species-specific TE library identified in the first dnaPipeTE run to infer accurately the TE% coverage in the final dnaPipeTE library for each species. Additionally, these TE sequences were confirmed with the Censor webtool and Blastx. Gene families and satellites were removed as described above, and the classification of TEs was confirmed using the Censor webtool. The resulting TE libraries from unassembled genomes were added to the Trypanosomatid TE DataBase v. 1.0.

### Estimation of the relationship between genome size and RE abundance

The abundances of RE and TEs vs genome assembly size were used for the correlation tests after the data were transformed to a logarithmic scale using tidyverse in R v. 4.2.1 (Wickham, 2016). To estimate correlation and the Spearman rank sum, with alpha = 0.005 (*lm* method), we used the Ape package in phytools 2.0 (Revell, 2024) and ggplot2 in R (Paradis and Schliep, 2019). We applied the Spearman rank correlation test as our data does not follow the normal distribution, as assessed by the Kolmogorov–Smirnov (KS) test. Additionally, using linear regression equations, we further inferred the relationship between genome size and the aforementioned traits with the Hiplot web tool (Li et al., 2022). Lastly, we also applied a phylogenetically independent contrasts (PICs) method (Felsenstein, 1985) to test a probable phylogenetic effect over the correlation among TEs/RE and genome size by using the pic function of Ape.

### Phylogenetic analysis of the family Trypanosomatidae

We employed a set of genes recovered through BUSCO analysis to infer the species tree. We extracted 40 single-copy genes from all the 57 trypanosomatid species from the GenBank [accessed 07/15/2024] (Supplementary Table S1). To validate the accuracy of the orthologs, we ran OrthoFinder v. 2.5.4 with the default settings (Emms and Kelly, 2019). The resulting proteins from the orthologous groups were aligned using MAFFT v. 7.453 with the auto option (Katoh and Standley, 2013), followed by the trimming step in TrimAl v. 1.4 (Capella-Gutiérrez et al., 2009) to remove gaps using -option ‘automated1.’ Next, the alignments were concatenated using FASconCAT v. 1.04 (Kück and Longo, 2014) to build a supermatrix of sequences from the 57 species, resulting in an alignment of 24 659 amino acids in length and 3.7% missing data. The best substitution model was automatically selected by the ModelFinder with the MFP option (Kalyaanamoorthy et al., 2017). A maximum likelihood (ML) tree was inferred using IQTree2 v. 2.0.4 with 100 bootstrap samples, and later on 1000 UFBS (ultra-fast bootstrap) with default options (Minh et al., 2020). Additionally, we ran RAxML-NG v. 1.1.0 with 100 bootstrap samples to compare topologies (Kozlov et al., 2019). By employing 2 likelihood-based phylogenetic inference tools we aimed to uncover potential disparities in tree topologies. Finally, the phylogenetic trees were rendered in the iTOL v. 6 (Letunic and Bork, 2024) web server and further refined with Inkscape v. 0.92.5 to add the status (presence/absence) of each TE clade based on the analyses described above.

## Results

### Trypanosomatidae species representation, genome quality and TE library construction

Recently, the taxonomy of the family Trypanosomatidae has undergone a revision resulting in a system with 7 subfamilies and 24 genera (Maslov et al., 2019; Kostygov et al., 2024). In light of this, our dataset included 57 species representing 19 genera (Supplementary Figure S3; Supplementary Table S1). Highlighting 2 medically important genera, there were 25 *Leishmania* spp. belonging to all 4 subgenera (*Leishmania, Mundinia, Sauroleishmania* and *Viannia*), and 9 *Trypanosoma* spp. We also used 4 species whose genome sequences were obtained in our laboratory, namely *Lafontella mariadeanei, Herpetomonas samuelpessoai, Sergeia* sp. (isolate 2467) and *Blechomonas* sp. (isolate 303E). Most species belonging to *Leishmania* genus had high BUSCO values (Supplementary Figure S4), except for *Leishmania lainsoni*, which had 10 fragmented genes. The BUSCO scores were similar to those reported for *L. major* (Friedlin), which serves as a benchmark for completeness (Camacho et al., 2021). Missing genes were documented in *Blastocrithidia nonstop* (7), *Trypanosoma b. equiperdum* (7), *Trypanosoma congolense* (5), *H. samuelpessoai* (5), *Kentomonas sorsogonicus* (4) and *L. mariadeanei* (1) (Supplementary Figure S4).

**Figure 2.**
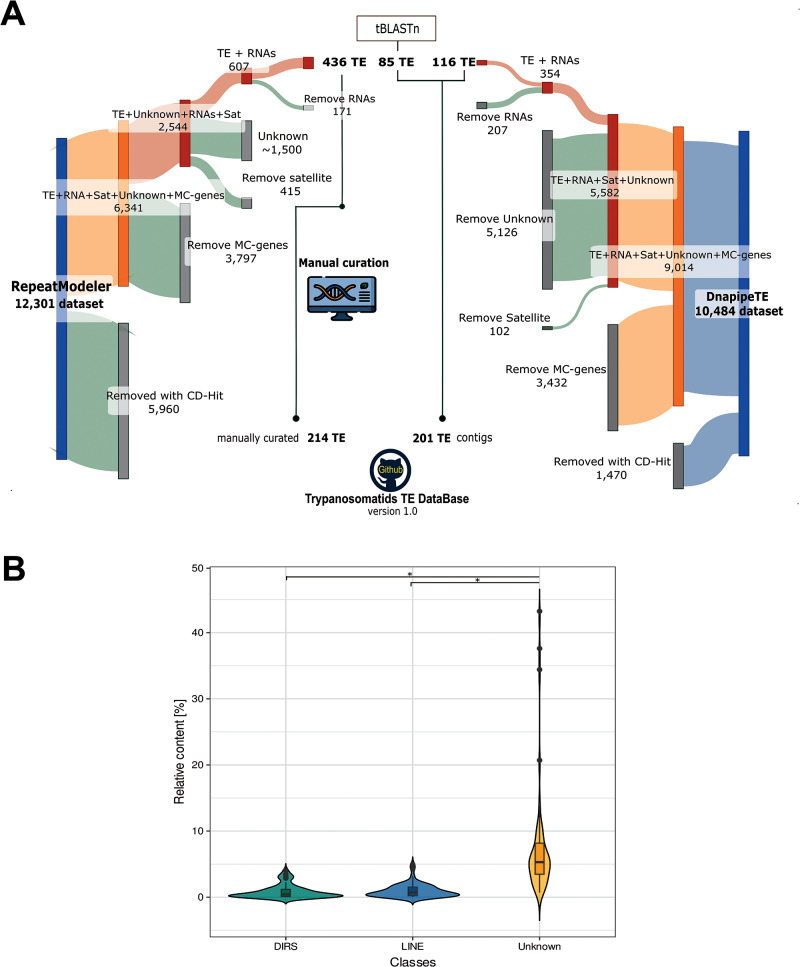
Overview of the steps to curate the final TE database and comparison among RE types. (A) Sankey plot displaying the raw RE libraries built by RepeatModeler (RM) and dnaPipeTe pipelines. For each phase, the grey portion indicates the number of consensus models removed by the filtering process, while the coloured segment depicts the number that continued to the next step. (1) Initial clustering reduced the number of family copies (5960 for RM and 1470 for dnaPipeTE) to streamline curation and minimize redundant TE models. (2) Multicopy genes (3797 for RM and 3432 for dnaPipeTE) were identified and removed using a homology-based approach. (3) Potential satellite sequences (415 for RM and 102 for dnaPipeTE) and ‘unknown’ sequences (1500 for RM and 5126 for dnaPipeTE) were excluded. (4) RNA-related families (171 for RM and 207 for dnaPipeTE) were detected and separated from the TE dataset. Following this pipeLINE, 436 TE sequences were manually curated, resulting in 214 canonical TE models. additionally, a small number of TEs were incorporated based on Tblastn results. (B) Violin plot representing the genome occupancy of 2 TE orders as DIRS, LINE, along with unknown from 57 assessed trypanosomatid genomes. Pairwise Wilcoxon rank test with Bonferroni correction was used for comparison among classes, where * (*P*-value < 0.01) indicates significance (Supplementary Table S3).

To provide a comprehensive overview of TEs in the family Trypanosomatidae, we ran RepeatModeler and dnaPipeTE pipelines across 37 genome assemblies and 20 unassembled short-read libraries, respectively. As a result, we obtained 12 301 consensus sequence models with RepeatModeler, while dnaPipeTE retrieved 10 484 contig models. The consensus models depicted the overview of REs. Because we focused solely on TEs, the raw repeat libraries underwent several filtering steps, to remove potential false positives (Figure 2A).

From the RepeatModeler approach, we recovered a set of 436 sequences with predicted TE-related proteins using Blastx. Additionally, some TEs were recovered using Tblastn, adding 85 model sequences. We also tried to classify the sequences annotated by RepeatModeler as ‘unknown’ (∼1500 sequences). Several of them were discarded because they represented additional multicopy genes or tandem repeats, while a few were confirmed as TEs. The remaining sequences lacked any traits of TEs or similarity to known TEs. We chose to retain only sequences with confirmed classification in the final TE library, totalling 214 TE families (Figure 2A). As expected, the sequences recovered from dnaPipeTE were TE contigs (very fragmented), and, after the filtering steps, only 116 TE contigs were confirmed.

From all REs found, we confirmed only 4 previously known trypanosomatid TE clades (*INGI, CRE, VIPER* and *TATE*). A few potential TEs classified as DNA transposon (*Helitron*) and LTRs (*Gypsy* and *Copia* elements) were not confirmed after the curation steps, as they were confirmed to be multicopy genes (false positives). Notably, the majority of the REs found were classified as unknown (Figure 2B).

### RE and TE content of trypanosomatid genomes

**Figure 3.**
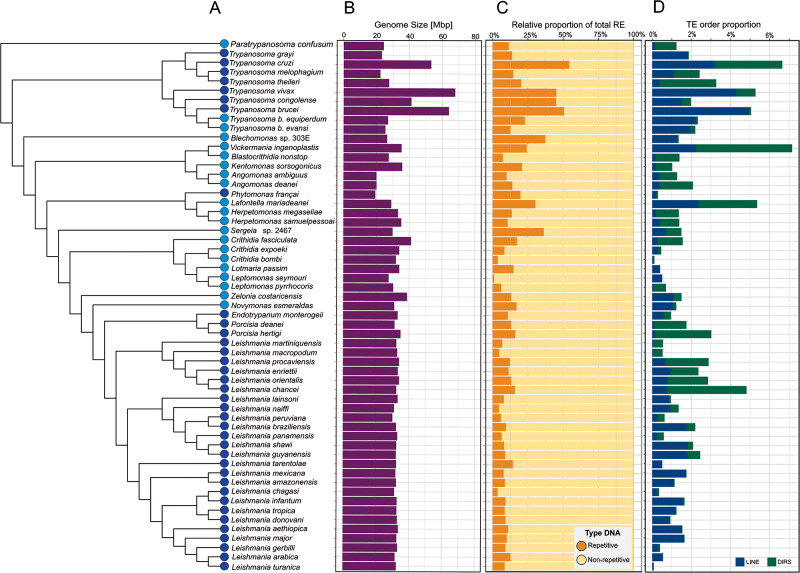
Contribution of REes and TEs to trypanosomatid genomes. (A) A cladogram displays the relationship of the 57 trypanosomatid species of 7 subfamilies used in this study. Monoxenous and dixenous parasites are marked by light and dark blue circles, respectively. (B) Genome sizes are shown in Mbp. (C) Proportion of repetitive and non-repetitive content in each species. (D) Total proportion of each TE order, DIRS (green) and LINE (blue).

The proportion of RE (multicopy genes, unknown, RNAs) and TE content was mapped onto the phylogenetic tree of trypanosomatids (Figure 3A; Supplementary Figure S5). The genome size of trypanosomatids varies widely, ranging from ∼20 Mbp in *Angomonas deanei* and *Phytomonas françai* to 67 Mbp in *Trypanosoma vivax* (Figure 3B; Supplementary Table S4). The genus *Leishmania* displays a lesser variation in length, ranging from ∼30 to ∼35 Mbp. Overall, the haploid genome size in trypanosomatids was spread around the mean of 33.0 Mbp with a standard deviation (s.d.) of 8.3 Mbp.

The repetitive content also varies widely among the trypanosomatid taxa (Figure 3C, Supplementary Table S4) from 3.7% (*Crithidia bombi*) to 56.1% (*T. cruzi*) with a mean of 15.2% (s.d. of 11.9%). As expected because of their pivotal role, rRNA and snoRNAs gene families were detected among the RE across all genomes. Moreover, the multicopy gene families represent the most abundant elements in the interspersed repeats fraction of the trypanosomatid genomes, ranging from 44.1% in *T. cruzi* to 0.7% in *C. bombi* (Supplementary Figure S5; Supplementary Table S4).

In terms of TE proportions, trypanosomatids presented a mean of 1.8% (s.d. of 1.6%) with most species having less than 3% of their genomes present as TEs. Higher proportions of TEs were found in *Vickermania ingenoplastis* (7.2%), *T. cruzi* (6.7%), *Lafontella mariadeanei* (5.4%), *T. vivax* (5.4%), *T. brucei* (5.0%), *Leishmania chancei* (4.8%), *Trypanosoma theileri* (3.3%) and *Porcisia hertigi* (3.0%). Conversely, a lower proportion was documented in *C. bombi* with ∼0.1% (Figure 3D; Supplementary Table S4). Notably, we detected greater proportions of TEs in some species of the subgenus *Mundinia*, including *L. chancei, Leishmania procaviensis* (2.9%), *Leishmania orientalis* (2.9%) and *Leishmania enriettii* (2.3%), but with the exception of *Leishmania macropodum* (0.1%).

Our results show that LINEs are more widely distributed than DIRS, and their proportions vary among the genomes (Figure 3D; Supplementary Table S4) comprising up to 4.9% in *T. b. brucei*, 4.3% in *T. vivax*, and 3.2% in *T. cruzi* genomes. In contrast, their proportions in *L. macropodum* and *L. martiniquensis* accounted for ∼0.02%. The DIRS elements were present at a higher proportion in *L. chancei* (4.1%) and *T. cruzi* (3.5%) and were either absent or present at a low proportion (0.01%) in some *Leishmania* spp.

This study is the first to report the TE proportions in several trypanosomatid species, with some showing relatively high values not previously reported for trypanosomatid species: *T. b. equiperdum* (2.3%), *T. vivax* (5.4%), *Trypanosoma melophagium* (2.4%), *L. mariadeanei* (5.4%), *B.* nonstop (1.4%), *L. guyanensis* (2.5%), *L. shawi* (2.2%), *L. lainsoni* (0.9%) and *V. ingenoplastis* (7.2%).

### Correlation test of REs and TEs vs trypanosomatid genome size

**Figure 4.**
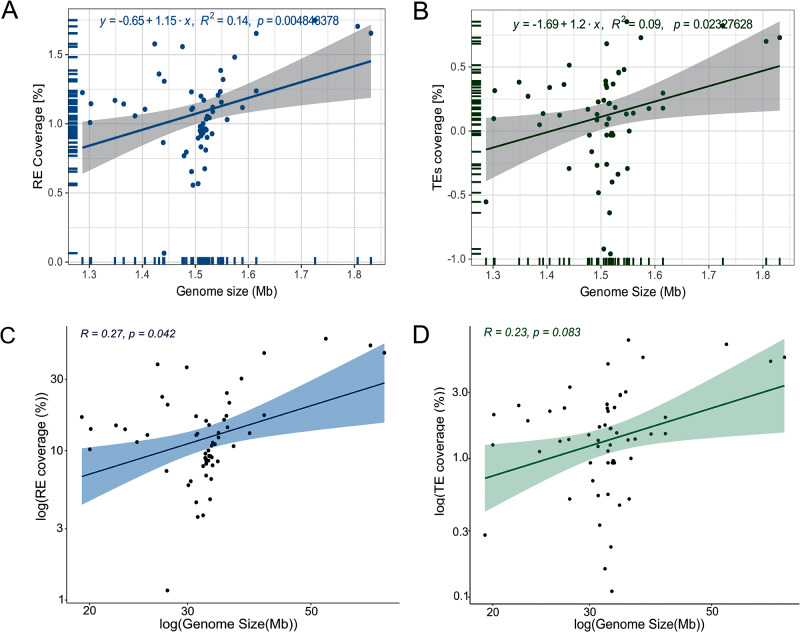
Scatterplots of correlation between genome size vs REs and TEs [%] across 57 trypanosomatid genomes. (A) Linear regression plot between genome size and the percentage of REs. (B) Linear regression plot between assembly genome size and the percentage of TEs. (C) Correlation plot between genome size and the percentage of REs. (D) correlation plot between genome size and the percentage of TEs. Lines: linear regression, shaded area: confidence interval.

The differences in trypanosomatid genome size prompted us to inquire how much REs and TEs contribute to this trait. The linear regression model revealed that RE and TE coverages have a weak correlation with the genome sizes (*R*^2^ = 0.14, *P* = 0.00484; *R*^2^ = 0.09, *P* = 0.02327, respectively) (Figure 4A, B). We confirmed that our data does not follow the normal distribution based on KS statistics (KS = 0.247, *P* = 0.0014). Additionally, the Spearman rank correlation test revealed a significant, albeit modest, positive correlation between the abundance of all REs and genome size (Spearman’s rank sum test *rho* = 0.27, *P* = 0.042) (Figure 4C). In contrast, the correlation between genome size and TEs was not statistically significant (Spearman, *rho* = 0.23, *P* = 0.083) (Figure 4D). We further tested PICs to correct for the non-independency of traits among species. These tests revealed significative relationships of genome size with RE (*R*^2^ = 0.5316, *P* = 7.639 × 10^−11^) and TEs (*R^2^* = 0.4897, *P* = 8.367 × 10^−10^) (Supplementary Figure S6).

### Distribution of TEs across the family Trypanosomatidae

In the last decade, molecular phylogenetic analyses have significantly enhanced our understanding of the extended relationships within the family Trypanosomatidae, providing valuable insights into the evolutionary processes in this group (Yurchenko et al., 2016; Kostygov and Yurchenko, 2017; Espinosa et al., 2018; Kaufer et al., 2019; Kostygov et al., 2020). In this sense, our study contributed to extending this analysis by including newly sequenced genomes of *Lafontella mariadeanei, Herpetomonas samuelpessoai, Blechomonas* spp*. Sergeia* spp. along with *L. shawi* and *L. guyanensis*.

**Figure 5.**
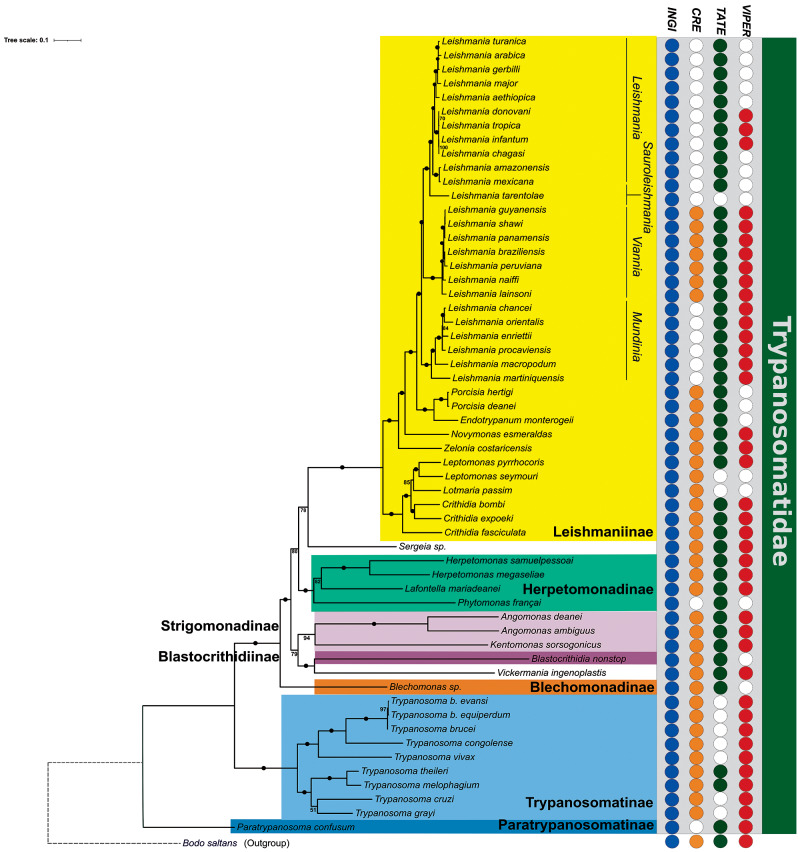
Phylogenetic relationships across 57 species of trypanosomatids. The bold letters show the position of the 7 subfamilies that currently constitute the family Trypanosomatidae. Bootstrap supports are shown at nodes, and maximum bootstrap support (100%) is shown with black circles. The distribution of the 4 retrotransposon clades is shown on the right, with coloured circles indicating the presence and white circles indicating the absence of a given element. *Bodo saltans*, a free-living phagotroph, was added as an outgroup. The scale bar indicates the number of substitutions per site.

The ML method was applied to the supermatrix to recover the best and most robust trypanosomatid phylogenetic tree, as depicted in Figure 5. As expected, all the subfamilies formed well-supported clades (100%), but there were some exceptions, such as observed between the Leishmaniinae and *Sergeia* spp. clades, with a bootstrap support of 78%. Moreover, the clade that included *T. cruzi* and *T. grayi* had a low bootstrap support (51%). Additionally, the RAxML tool showed a different topology for this clade (Supplementary Figure S7).

Understanding these phylogenetic relationships is essential to visualize the evolutionary pattern of the TEs (Figure 5). Notably, *Bodo saltans*, a free-living kinetoplastid species, is also known to harbour the 4 TE clades (*CRE, INGI, TATE* and *VIPER*), indicating that these elements were likely present in the last common ancestor of all trypanosomatids (Jackson et al., 2008, 2016; Ribeiro et al., 2019).

The *INGI* superfamily is the most widespread, found in all 57 lineages analysed with a number of copies varying from only a few in *Leptomonas pyrrhocoris* to 1671 in *L. aethiopica*. However, these abundant copies in Leishmania appear to be mostly non-autonomous (Table 1). In contrast, *CRE* clade showed a patchy distribution, with high numbers in *V. ingenoplastis, T. cruzi* and *L. mariadeanei*. Four independent events of loss could explain the distribution pattern of these elements: in *P. confusum*, in *P. françai*, in the ancestor of the subgenus *Mundinia*, and in the common ancestor of the subgenera *Leishmania* and *Sauroleishmania*.

*VIPER* and *TATE* also displayed a patchy distribution pattern. Here, we reported for the first time *VIPER* in multiple monoxenous genera and remnants in several *Leishmania* spp. High copy numbers in *T. cruzi, L. mariadeanei*, and *V. ingenoplastis* may reflect retained activity in these lineages. Moreover, we found *TATE* elements in all *Leishmania* spp. investigated (except *L. tarentolae*) with high loads in the *Mundinia* subgenus and *V. inglenoplastis*. In *Trypanosoma* spp., *TATE* has been previously detected only in *T. theileri* (Ribeiro et al., 2019). Here, we also identified this element in the genome of a closely related species, *T. melophagium*.

Our analysis revealed a highly variable number of TE copies across 37 assembled trypanosomatid genomes. However, it is important to recognize that these counts do not necessarily reflect complete or functional copies, as they may include remnant fragments. The copy numbers can also be influenced by the quality of the genome assemblies and TE libraries. For instance, we noticed that the TE consensus sequences from *Zelonia costaricensis* are fragmented due to the fragmentation of the genome itself (Tullume-Vergara et al., 2023), potentially leading to overestimation of the copy numbers. On the other hand, the *CRE* elements could be underrepresented in certain species if the SL-RNA region, where these elements typically insert, is not well-covered in the genome assembly. Therefore, while this provides an overall view of TE load across species, these findings should be interpreted with caution.

### TE transposition activity during trypanosomatid evolution

The K2P-based copy divergence analysis was performed to assess the diversity and dynamics of the trypanosomatid mobilome in detail. The TE landscapes illustrate the distribution of genome coverage of copies for LINE and DIRS sequences relative to their divergence from the consensus model sequence. The shape of a distribution landscape can be categorized as follows: (i) recent events of TE activity (transposition bursts) are characterized by low divergence scores (<5% divergence from the consensus) and are depicted with L-shaped peaks (Barrón et al., 2014); (ii) bimodal (2 peaks) and (iii) ‘bell-shaped’ curves depict an equilibrium between transposition and excision events over evolutionary time (Le Rouzic and Capy, 2005).

**Figure 6.**
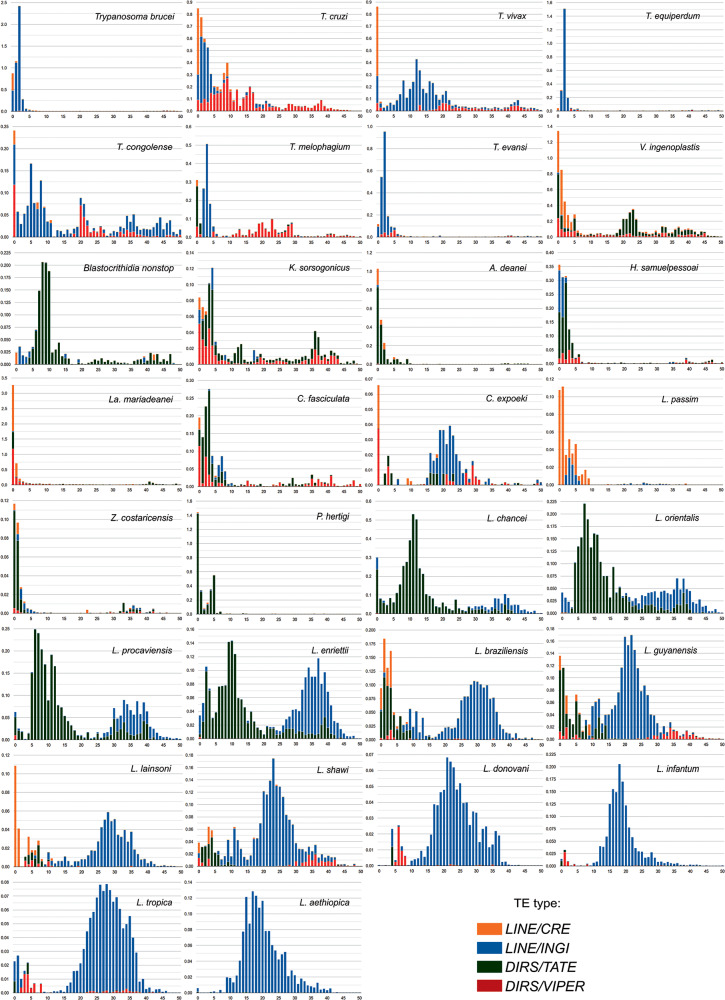
TE age distribution in trypanosomatid genomes based on K2P divergence analysis. The *y*-axis displays the percentage of the genome (abundance) for different clades of TEs, and the *x*-axis shows the Kimura substitution level (*k*-value from 0 to 50) of copies with their respective consensus sequences. Likewise, a low degree of divergence indicates recent activity (<5%), whereas higher divergence scores suggest that the copies derive from older transposition events.

The TE landscape distribution for 30 trypanosomatid genomes was compared among genera and species to describe some main events (Figure 6; Supplementary Figure S8). The *CRE* superfamily has acquired the majority of copies relatively recently (compared to the other retrotransposons) in almost all genomes possessing these elements. Very recent activity of this superfamily (including some notable bursts) can be seen in *H. samuelpessoai, L. mariadeanei, L. braziliensis, L. guyanensis, L. lainsoni, Lotmaria passim, T. brucei, T. congolense, T. cruzi, T. melophagium, T. vivax* and *V. ingenoplastis.* The other TE families exhibit different evolutionary trajectories depending on the species.

Within the genus *Trypanosoma*, the landscape distributions generally show a multimodal shape with *INGI* and *VIPER* being well represented. As expected due to their phylogenetic proximity, *T. brucei, T. b. equiperdum* and *T. b. evansi*, show a similar pattern with a very low number of ancient insertions, and a recent activity peak of *INGI* (K2P of 3) and *CRE* (K2P 0), although the *CRE* peak is more prominent in *T. brucei, T. congolense, T. cruzi* and *T. vivax* have a recent activity peak of *CRE, VIPER* and *INGI* and additional, more ancient peaks (K2P 9 and 16 in *T. cruzi*; K2P 12 in *T. vivax*; K2P 5 and 20 in *T. congolense*). Interestingly, in contrast to other *Trypanosoma* spp., *T. melophagium* does not present a very recent peak of *INGI* elements. On the other hand, in this species, the *TATE* elements appear to be the most recently active TEs, followed by the *VIPERs* and *CREs*.

The divergence landscape for *V. ingenoplastis* and *K. sorsogonicus* displayed a multimodal distribution with a significant proportion of sequences presenting divergence below 5%, which suggests a recent activity for the *CRE* and *VIPER* clades. *Blastocrithidia nonstop* showed a multimodal shape, with the first peak occurring earlier (from 10 to 5 of K2P), being dominated by *TATE* elements. This pattern of *B. nonstop* is likely to be associated with the accumulation of TEs in its genome.

Strikingly, we observed an L-shape distribution for *A. deanei, C. fasciculata, H. samuelpessoai, L. mariadenaei, L. passim, Porcisia hertigi* and *Z. costaricensis* with increasing trend spanning from ∼10% to 0% of K2P divergence. This pattern indicates a more recent burst of activity with a very low quantity of older copies in some of these species. The *CRE, TATE* and *VIPER* elements were well noticeable in *H. samuelpessoai* and *L. mariadenaei*. The *TATE* elements are the most abundant in *A. deanei, P. hertigi* and *Z. costaricensis* genomes, while *TATE* and *CRE* stand out in *L. passim*.

Within the genus *Leishmania*, similar patterns can be observed for the species belonging to the same subgenera. In the subgenus *Mundinia*, which includes *L. chancei, L. enriettii, L. orientalis* and *L. procaviensis*, multipeaked distributions of *TATE* and *INGI* were observed. The *TATE* elements are highly abundant in this group although this expansion was primarily due to some more ancient events of transposition (highest peaks of K2P 5-10). Furthermore, in subgenus *Viannia*, bimodal peaks were observed in *L. braziliensis, L. guyanensis, L. lainsoni* and *L. shawi* (although less pronounced in the latter). Recent activity of *CRE* and *TATE* elements is indicated for almost all these species, while *INGI* elements presented more ancient activity. Lastly, the TE landscape in the *Leishmania* subgenus is dominated by the *INGI* clade exhibiting an ancient peak, similar to what is observed in the subgenera *Mundinia* and *Viannia*. This pattern is expected, given that only non-autonomous *INGI*-related elements (SIDERs and DIREs) are present in the genomes of *Leishmania* spp. Interestingly, *L. donovani, L. infantum* and *L. tropica* still contain remnants of the *VIPER* elements, reported for the first time in this study.

Unexpectedly, a low percentage of copies with very low divergence (K2P 0) is observed despite the absence of active TEs. Noteworthy, this could suggest that some TEs are being duplicated through mechanisms other than transposition, such as through segmental genomic duplications. This possibility could help explain the persistence of non-autonomous TEs even when active elements are absent and merits further investigation. In addition, some of these observations could result from false duplications in the assemblies. Even applying, purging steps, such artefacts are known to occur (Ko et al., 2022).

For several species, we observe a very low amount of ancient elements. Possible explanations include (1) loss of active TEs followed by a recent invasion of active families from other species, although no evidence of horizontal TE transfer has been documented for these species; and (2) continuous production of new copies by active TEs with rapid turnover, which may lead to the elimination of older, inactive copies.

## Discussion

### Trypanosomatid TE database 1.0: A curated high-quality resource for future research

In recent years, the volume of trypanosomatid genomic data has increased dramatically. While the study of TEs is crucial for understanding the evolutionary dynamics and functionality of genomes, they are often analysed superficially. Our work significantly extended the understanding of the trypanosomatid mobilome through a comparative analysis encompassing 57 genomes that included new non-model trypanosomatids. It revealed TE abundance, diversity, activity and evolution.

To ensure the reliability of outcomes, we executed 2 pipelines based on repetitiveness, RepeatModeler and dnaPipeTE. The latter prevents underestimation of the TE proportion, a common problem for genome assemblies based on short reads as they can be fragmented and present collapsed contigs (Alkan et al., 2011; Peona et al., 2018; Shahid and Slotkin, 2020).

The TE prediction approach employed in this work also addressed common issues associated with similarity-based methods. Specifically, the TE databases often harbour a low representation of curated models for non-model microorganisms and, consequently, rely solely on similarity-based methods, which can limit the detection of TEs in these underrepresented or novel species (Storer et al., 2021). On the other hand, some TEs might be missed by repetitiveness-based methods due to their insufficient repetitiveness and, therefore, we complemented our analyses with BLAST-based searches.

Confirming sequences as genuine TEs and classifying them correctly is crucial but complex, as programs like RepeatModeler can detect a wide range of repetitive DNAs and sometimes misclassify sequences (Almutairi et al., 2021a). Distinguishing between the total repetitive content of the genome (RE) and the TE content is critical in any discussion concerning transposons. Moreover, since trypanosomatid genomes exhibit low diversity (Bringaud et al., 2008), new TEs identified by such programs require careful scrutiny. In this study, manual curation ensured accurate TE classification. Additionally, while we attempted to classify unknown sequences, most remained unclassified. Although some of them might be genuine TEs, the absence of typical protein domains and TE structures suggests they are unlikely to be conventional elements, making their characterization particularly challenging.

In this work, after a concerted effort to overcome the challenges in characterizing trypanosomatid TEs, we ultimately confirmed 214 consensus TE families across 37 species. These curated sequences are now part of the Trypanosomatid TE Database 1.0, presenting a valuable resource for future studies.

### RE and TE content in trypanosomatid genomes

Based on the first genomic sequences of trypanosomatids, significant variation in the proportion of the repetitive genomic content has been noted, with the vast majority of it allocated to the multigenic families (Ivens et al., 2005; Pita et al., 2019). Considering the total proportion of REs, our data agree with previous works for *C. fasciculata* (Albanaz et al., 2023), *T. cruzi* (Pita et al., 2019), and *L. major* and *L. martiniquensis* (Almutairi et al., 2021a; Albanaz et al., 2023). A higher proportion of REs than previously reported was found in *H. samuelpessoai* and *T. brucei* compared to previous reports (Berriman et al., 2005; Pita et al., 2019; Albanaz et al., 2023). According to our results, the highest repetitive contents are reported in the genus *Trypanosoma.*

Earlier studies suggested that TEs comprise up to 5% of the genomic content in trypanosomatids (Bringaud et al., 2007) while later analyses elevated this number to 12% (Pita et al., 2019). A recent work estimated that 48% of the *T. cruzi* genome are TEs (Hoyos Sanchez et al., 2024). However, this estimate was obtained with raw RepeatModeler libraries that likely included other repetitive sequences, such as the large gene families or unknown sequences. In our study, we report a lower proportion for *T. cruzi*, which can be explained by the different TE prediction tools used and the manual curation of consensus sequences. The highest proportion of TEs in this work was ∼7%, for *V. ingenoplastis.* Although this proportion is much smaller than what is found in mammals and plants, it is comparable to or slightly lower than what was found in some unicellular protists, such as *Entamoeba* sp. (5–8% (Pritham, 2009)), apicomplexans (up to 5.4% (Rodríguez and Makalowski, 2022)) and the amoebozoan *Dictyostelium discoideum* (∼10%; Glöckner et al., 2001). Multiple interconnected factors are known to contribute to TE abundance, including transposition activity, historical accumulation of TEs, silencing mechanisms, competition between TEs, occasional positive selection for beneficial insertions (Betancourt et al., 2024) and the strength of purifying selection acting against the TEs, which itself is affected by population size (Lynch and Conery, 2003; Betancourt et al., 2024).

### Phylogeny of trypanosomatid species

Our reconstructed phylogenetic tree was broadly consistent with previous reports based on various nuclear or kDNA markers (Kaufer et al., 2019; Maslov et al., 2019; Kostygov et al., 2021). The topology of the clade encompassing *T. cruzi* and *T. grayi* (with a low support in our work) is in line with the phylogenomics analysis of Kelly et al. (2014) that relied on a supermatrix of 959 single-copy nuclear genes. Moreover, in this work, we report the first multilocus-based analysis of the subfamily Herpetomonadinae. Similarly to the previous inferences based on 18S ribosomal RNA/gGAPDH sequences (Yurchenko et al., 2016), it placed *H. samuelpessoai* and *L. mariadeanei* as sister taxa.

### Diversity and evolution of TEs in Trypanosomatidae

To date, DNA transposons have not been reliably identified or characterized in trypanosomatid genomes; however, Merlin DNA transposons were recently discovered in the genomes of trypanosomatid-related *B. saltans* and *Perkinsela* sp. (Lopes et al., 2021). In this work, we found no evidence of any DNA transposons across the 57 analysed nuclear genomes. Thus, we could not confirm the presence of helitrons detected in the *L. martiniquensis* genome (Almutairi et al., 2021a) or other DNA transposons reported in *T. cruzi* (Hakim et al., 2024; Hoyos Sanchez et al., 2024), suggesting that they were false positives. Our findings corroborate the idea that trypanosomatid genomes are devoid of class II TEs. Considering that DNA transposons are present in the last common ancestor of kinetoplastids (Lopes et al., 2021), we propose that these elements were eliminated very early from the genomes of trypanosomatids. Our data support the hypothesis that the mobilome of a trypanosomatid ancestor was of low diversity and limited to the *INGI, CRE, TATE* and *VIPER*. This aligns with a recent report showing low TE diversity in the *Paradiplonema papillatum* genome, a species from a sister group to kinetoplastids (Valach et al., 2023).

Considering the distribution of the 4 TE clades, we conclude that these elements were generally effective in colonizing most trypanosomatid species. Nevertheless, some TEs were either ablated or degenerated during evolution. Events of complete TE loss occurred mostly independently either in single species (for example, *VIPER* and *TATE* in *Lotmaria passim* and *Leptomonas seymouri*) or in the ancestors of the species group (for example, *CRE* in *Mundinia*). The persistence of all 4 elements in several species across the family Trypanosomatidae suggests that either these elements were more active in certain species or their remnants were retained. This can be well exemplified by the *INGI* elements, which, despite the absence of active copies, were retained in the genomes as non-autonomous counterparts, likely due to their role in the control of gene expression (Bringaud et al., 2007; Heras et al., 2007).

Considering the mode of TE transmission, there have been no documented cases of horizontal transfer (HT) in trypanosomatids, despite the fact that this mechanism was reported for several trypanosomatid genes, such as catalase or proline racemase (Opperdoes and Michels, 2007; Caballero et al., 2015; Chmelová et al., 2021). In our dataset, we found no clear evidence of HT, such as the presence of unexpected elements that might represent acquisitions from non-trypanosomatid sources. Such acquisitions could be anticipated, given the ecological associations of these parasites with diverse host and vector species. HT of TEs has been observed in other systems with close ecological interactions; for example, in *Rhodnius prolixus*, a major vector of *T. cruzi*, multiple TE families have been identified as nearly identical to those found in its mammalian hosts (Gilbert et al., 2010; Schaack et al., 2010). While the possibility of TE lateral transfer among trypanosomatid species remains an area for further investigation, the apparent absence of this process raises important questions about the mechanisms that constrain TE transmission to a vertical model.

Despite manual curation of the presented database, we recognize that trypanosomatid TEs and their posited activities must be further characterized using wet-lab methods. Furthermore, future research could benefit from detailed phylogenetic analyses of the 4 trypanosomatid TE superfamilies to better understand their evolutionary trajectories and roles within the trypanosomatid genomes.

In this work, we provided the first in-depth report on the abundance and distribution of TEs in a large array of trypanosomatid species. We generated valuable custom TE libraries that can be employed by the trypanosomatid research community to improve the annotation of the mobilome for new genome assembly. Our comparative study provided new perspectives to understanding the events of gains and loss in the TE repertoire, elucidating the dynamics in trypanosomatid genome architecture.

## Supporting information

Tullume-Vergara et al. supplementary material 1 Tullume-Vergara et al. supplementary material

Tullume-Vergara et al. supplementary material 2 Tullume-Vergara et al. supplementary material

Tullume-Vergara et al. supplementary material 3 Tullume-Vergara et al. supplementary material

## Supplementary material

The supplementary material for this article can be found at https://doi.org/10.1017/S0031182025100231.
