# Supplementary material for: Comparative analysis of the mobilome yields new insights into its diversity, dynamics and evolution in parasites of the Trypanosomatidae family
Source: Parasitology. 2025 Jun 13;152(6):602–17. doi: 10.1017/S0031182025100231 (PMC12278014; doi:10.1017/S0031182025100231)
Supplement: Tullume-Vergara et al. supplementary material 1 — Tullume-Vergara et al. supplementary material [file S0031182025100231sup001.pdf]

Supplementary material

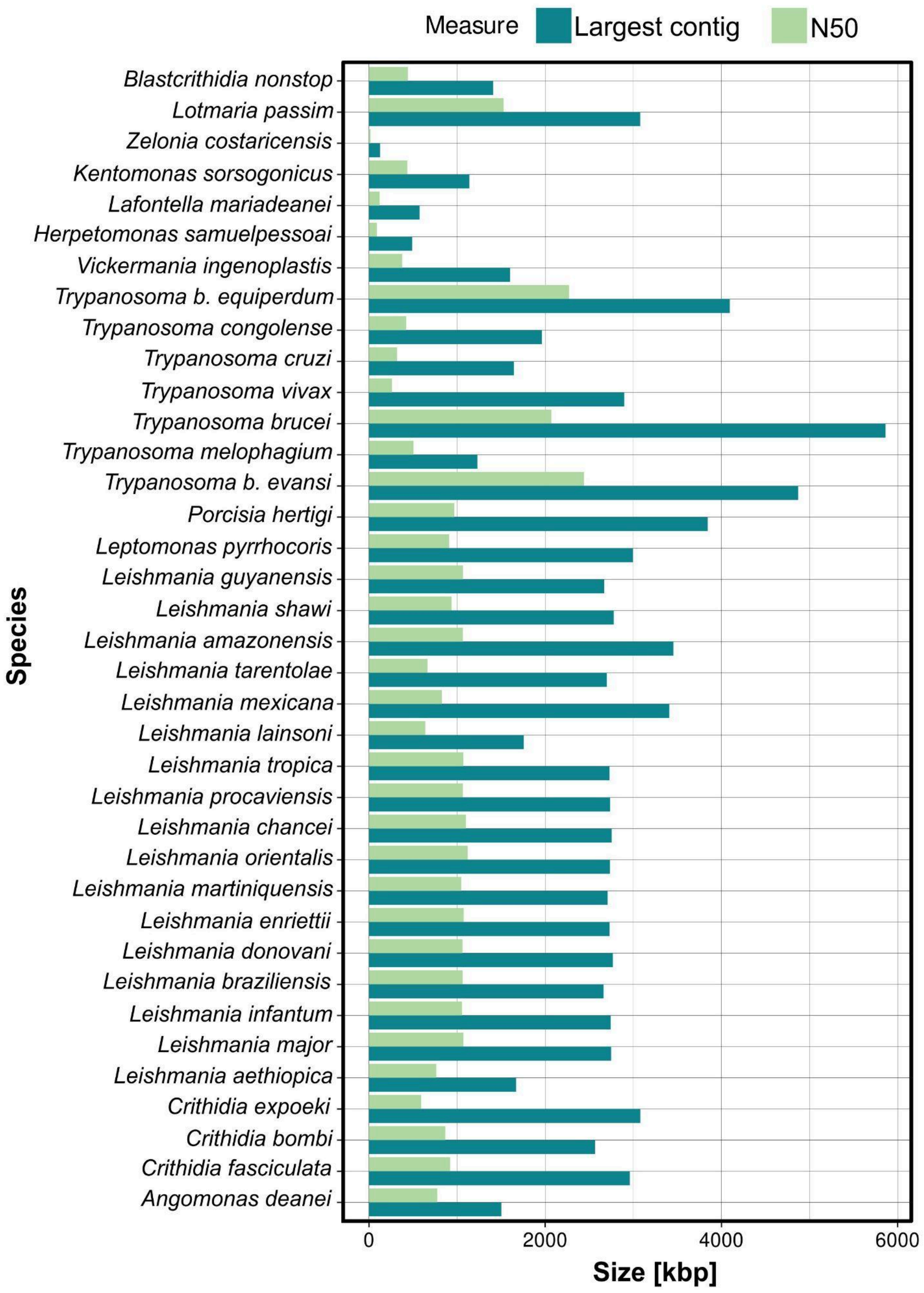

Figure S1. Statistical distribution of contig N50 and largest contig across 37 trypanosomatid genomes.

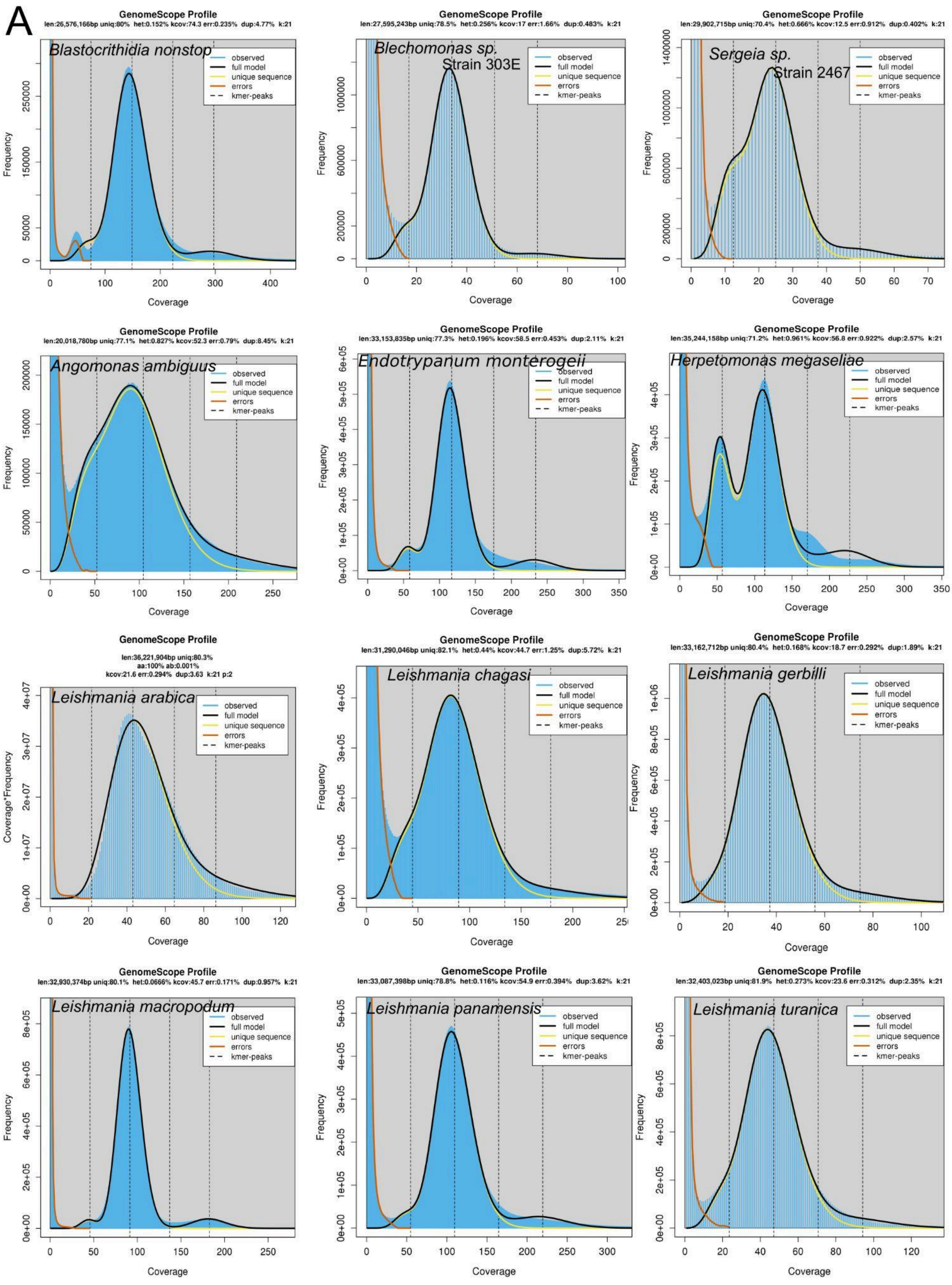

A
GenomeScope Profile
len:26,576,166bp uniq:80% het:0.152% kcov:74.3 err:0.235% dup:4.77% k:21
Blastocrithidia nonstop
GenomeScope Profile
len:27,595,243bp uniq:78.5% het:0.256% kcov:17 err:1.66% dup:0.483% k:21
Blechomonas sp.
Strain 303E
GenomeScope Profile
len:29,902,715bp uniq:70.4% het:0.666% kcov:12.5 err:0.912% dup:0.402% k:21
Sergeia sp.
Strain 2467
GenomeScope Profile
len:20,018,780bp uniq:77.1% het:0.827% kcov:52.3 err:0.79% dup:8.45% k:21
Angomonas ambiguus
GenomeScope Profile
len:33,153,835bp uniq:77.3% het:0.196% kcov:58.5 err:0.453% dup:2.11% k:21
Endotrypanum monterogeii
GenomeScope Profile
len:35,244,158bp uniq:71.2% het:0.961% kcov:56.8 err:0.922% dup:2.57% k:21
Herpetomonas megaseliae
GenomeScope Profile
len:36,221,904bp uniq:80.3%
aa:100% ab:0.001%
kcov:21.6 err:0.294% dup:3.63 k:21 p:2
Leishmania arabica
GenomeScope Profile
len:31,290,046bp uniq:82.1% het:0.44% kcov:44.7 err:1.25% dup:5.72% k:21
Leishmania chagasi
GenomeScope Profile
len:33,162,712bp uniq:80.4% het:0.168% kcov:18.7 err:0.292% dup:1.89% k:21
Leishmania gerbilli
GenomeScope Profile
len:32,930,374bp uniq:80.1% het:0.0666% kcov:45.7 err:0.171% dup:0.957% k:21
Leishmania macropodum
GenomeScope Profile
len:33,087,398bp uniq:78.8% het:0.116% kcov:54.9 err:0.394% dup:3.62% k:21
Leishmania panamensis
GenomeScope Profile
len:32,403,023bp uniq:81.9% het:0.273% kcov:23.6 err:0.312% dup:2.35% k:21
Leishmania turanica
observed
full model
unique sequence
errors
kmer-peaks
Frequency
Coverage*Frequency
Coverage

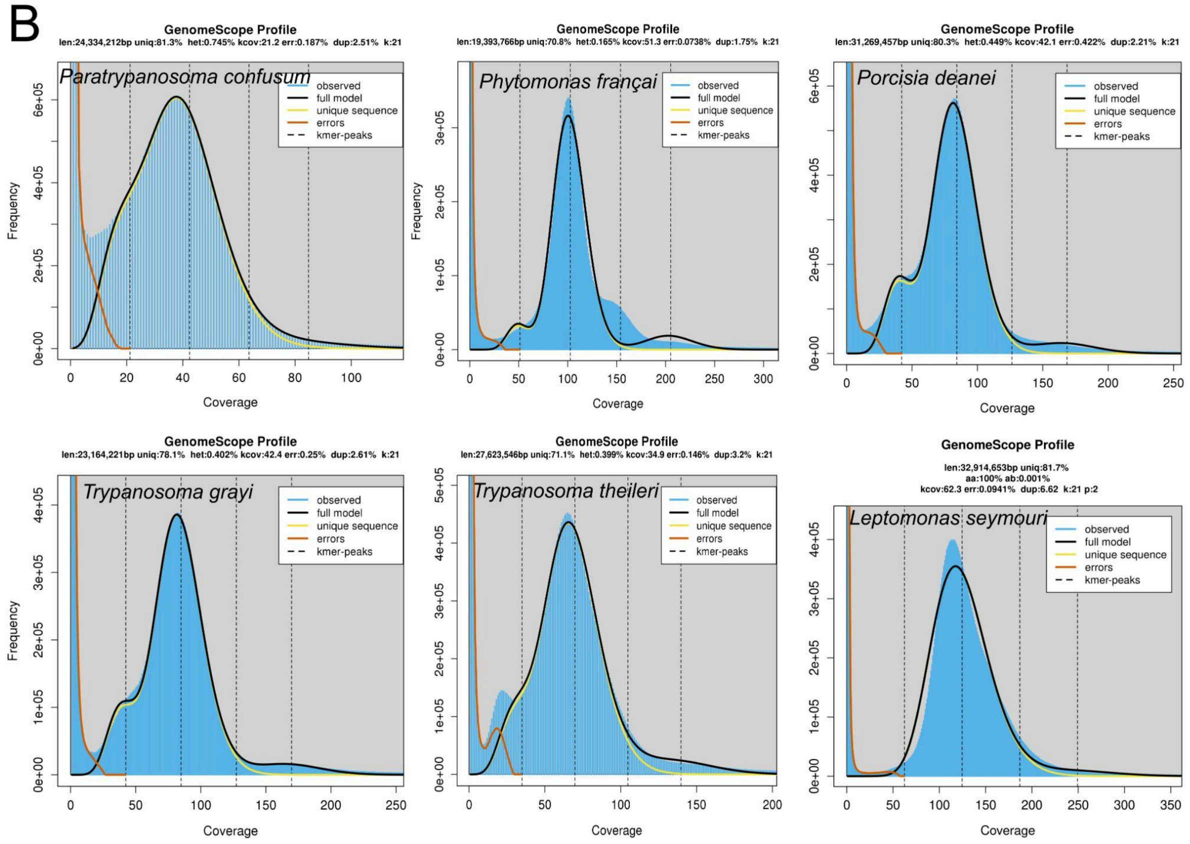

Figure S2. GenomeScope analysis of genome size, heterozygosity, and duplicate rate across 18 short-reads libraries. A) and B) The first peak corresponds to the heterozygous part and the second peak corresponds to the homozygous part. The Illumina short-read sequencing data was used to count *k*-mers in DNA by employing Jellyfish with K = 21.

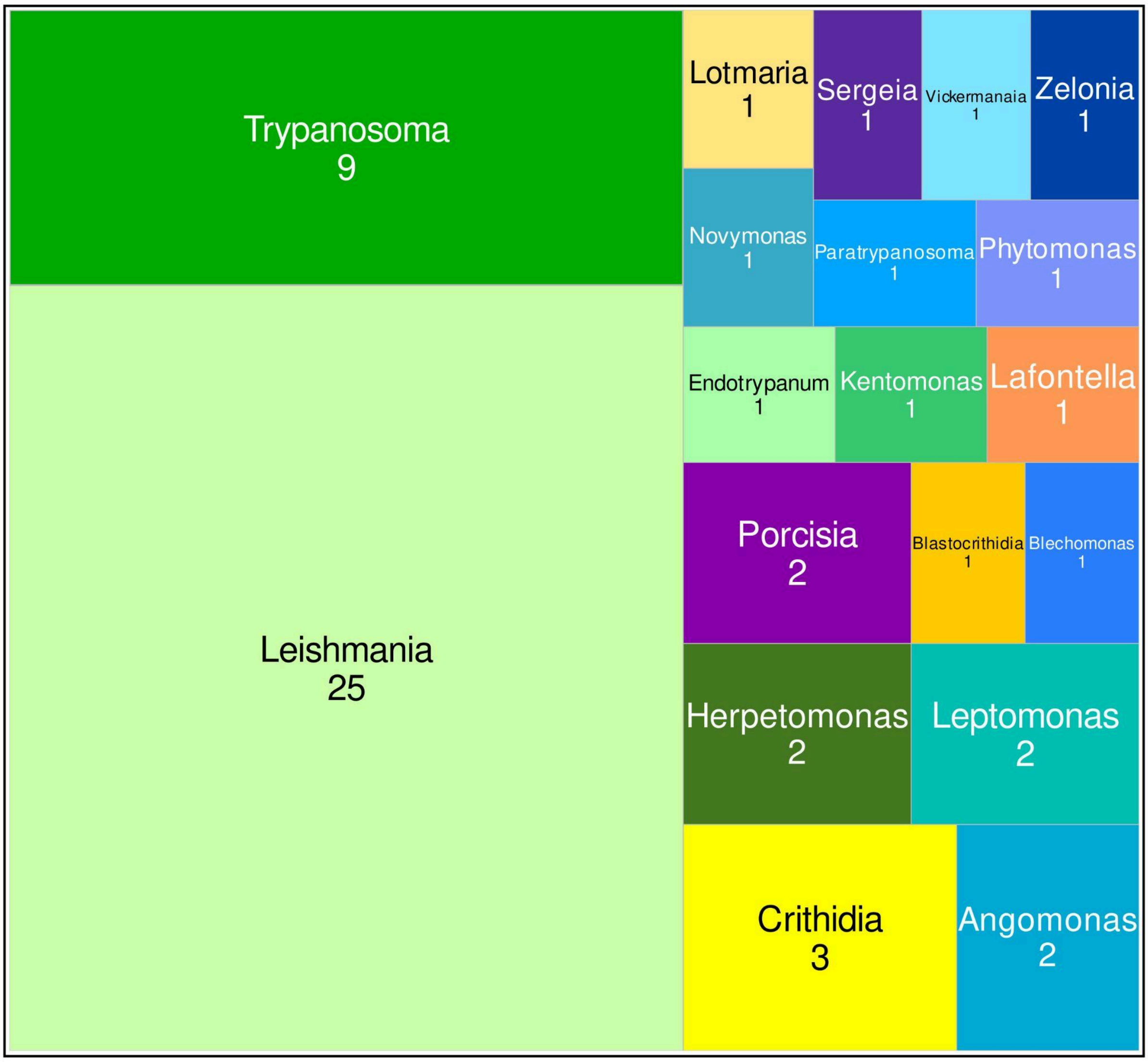

Figure S3. The genomics dataset was made up of 19 genera, highlighting *Leishmania* spp. (25 species) and *Trypanosoma* spp. (nine species).

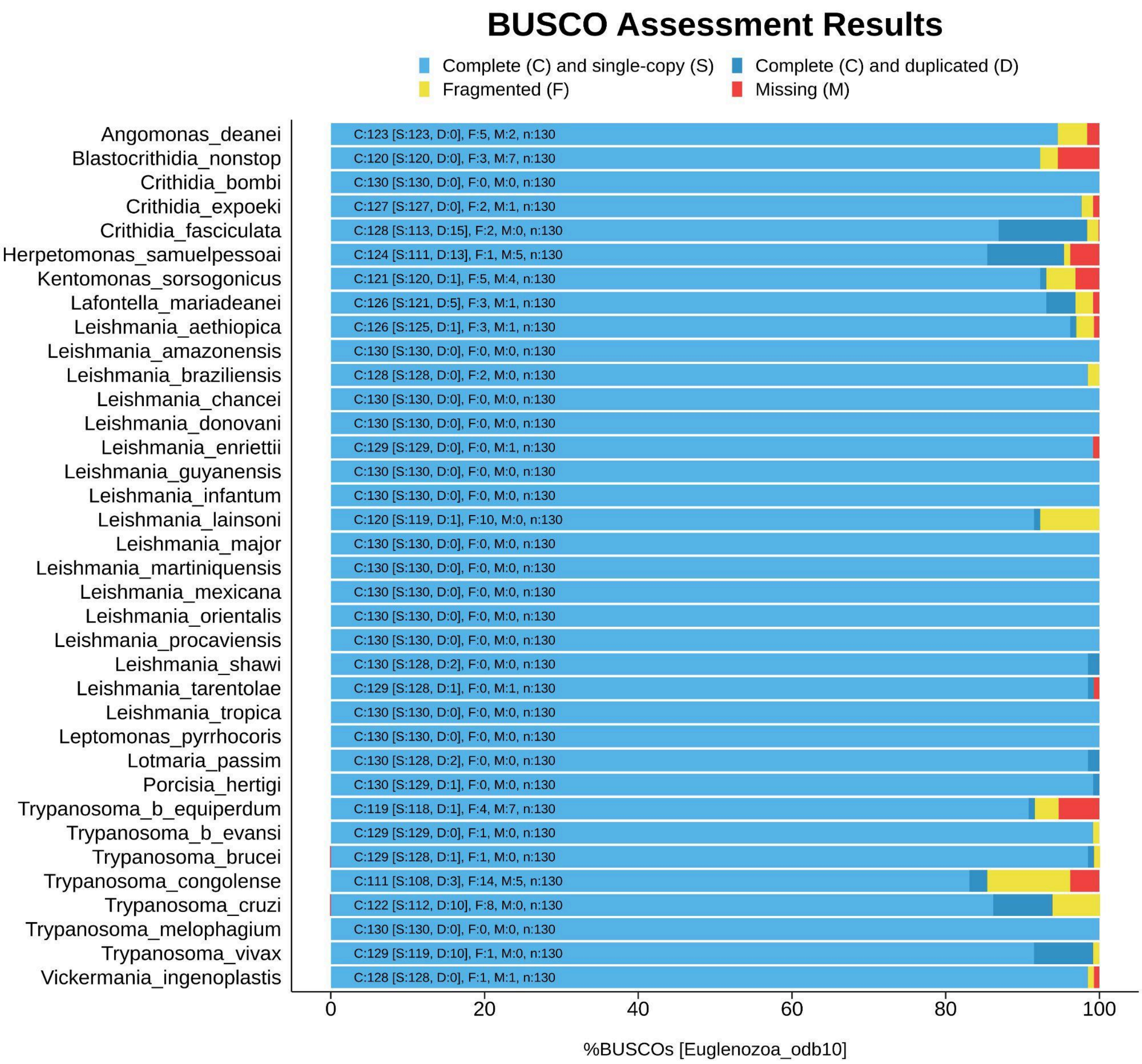

Figure S4. Bar plot showing the BUSCO completeness assessment of 36 genome assemblies (PacBio and Nanopore technologies). Proportion of BUSCO genes classified as: Single-copy orthologs (light blue), Duplicated (dark blue), Fragmented (yellow), and Missing (red).

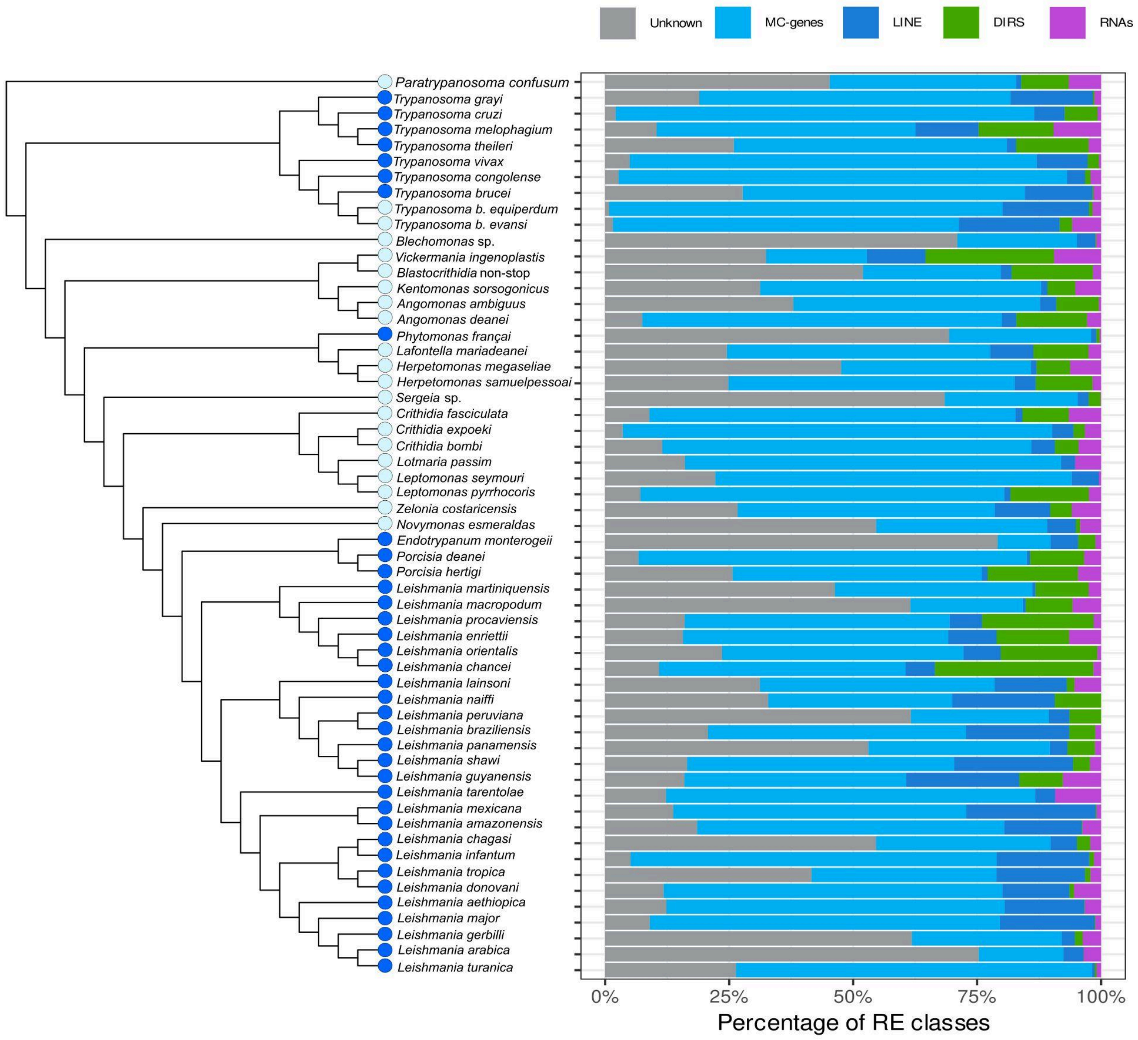

Figure S5. Phylogenetic relationships of 57 trypanosomatid species with their repeat class abundance.

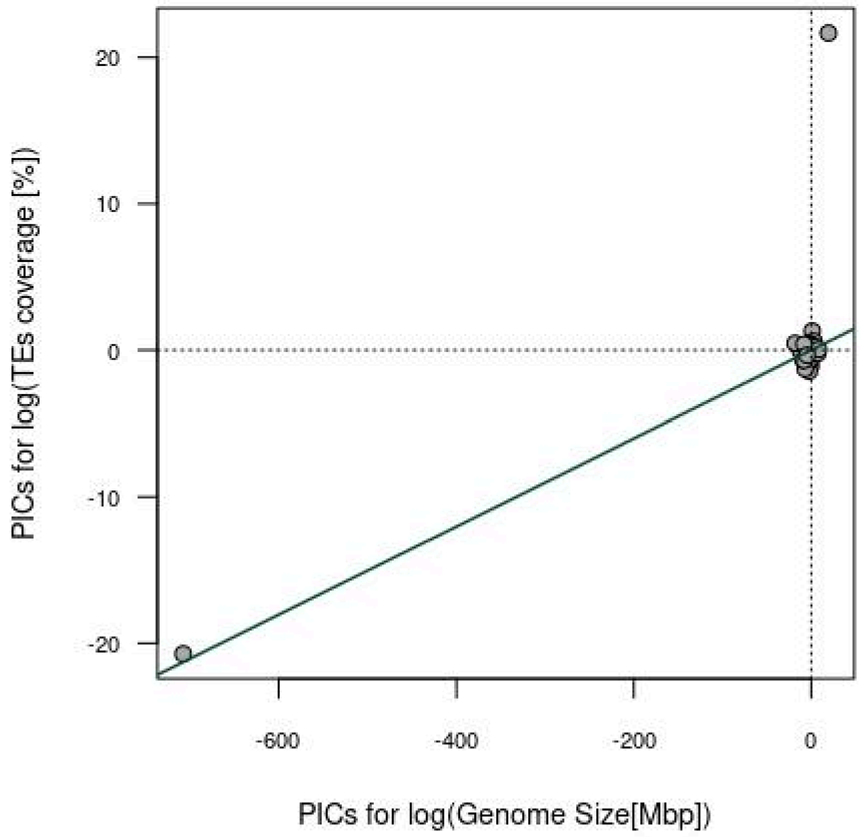

Figure S6. Correlation plot between assembly genome and the percentage of TEs supported by PIC (R^2 = 0.4897, p = 8.367 $\times 10^{-10}$). Lines: linear regression, green area: confidence interval.

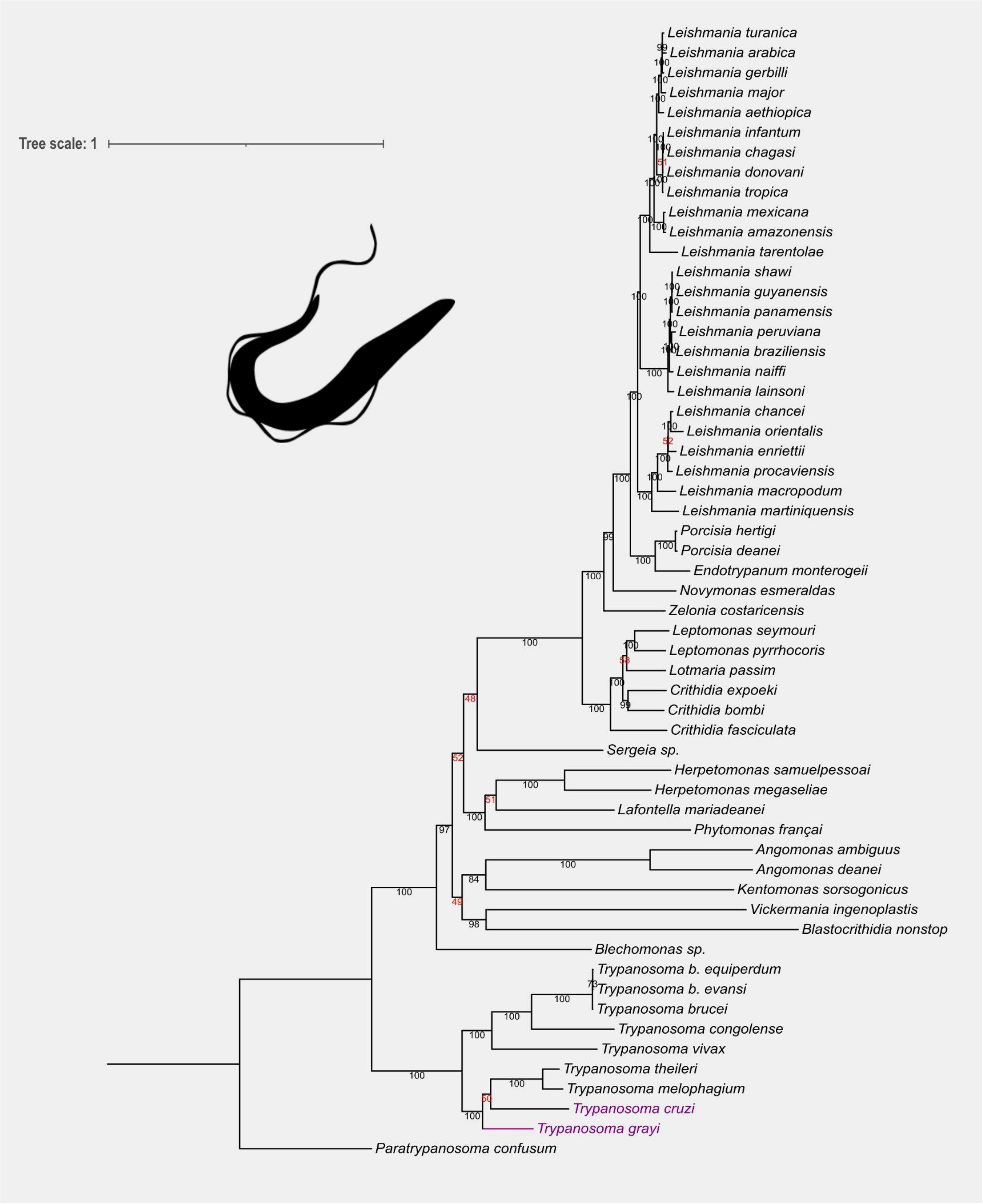

Figure S7. Maximum likelihood phylogenetic relationships across 57 species of trypanosomatids, obtained through the analysis of 40 concatenated BUSCO-derived orthologous proteins.

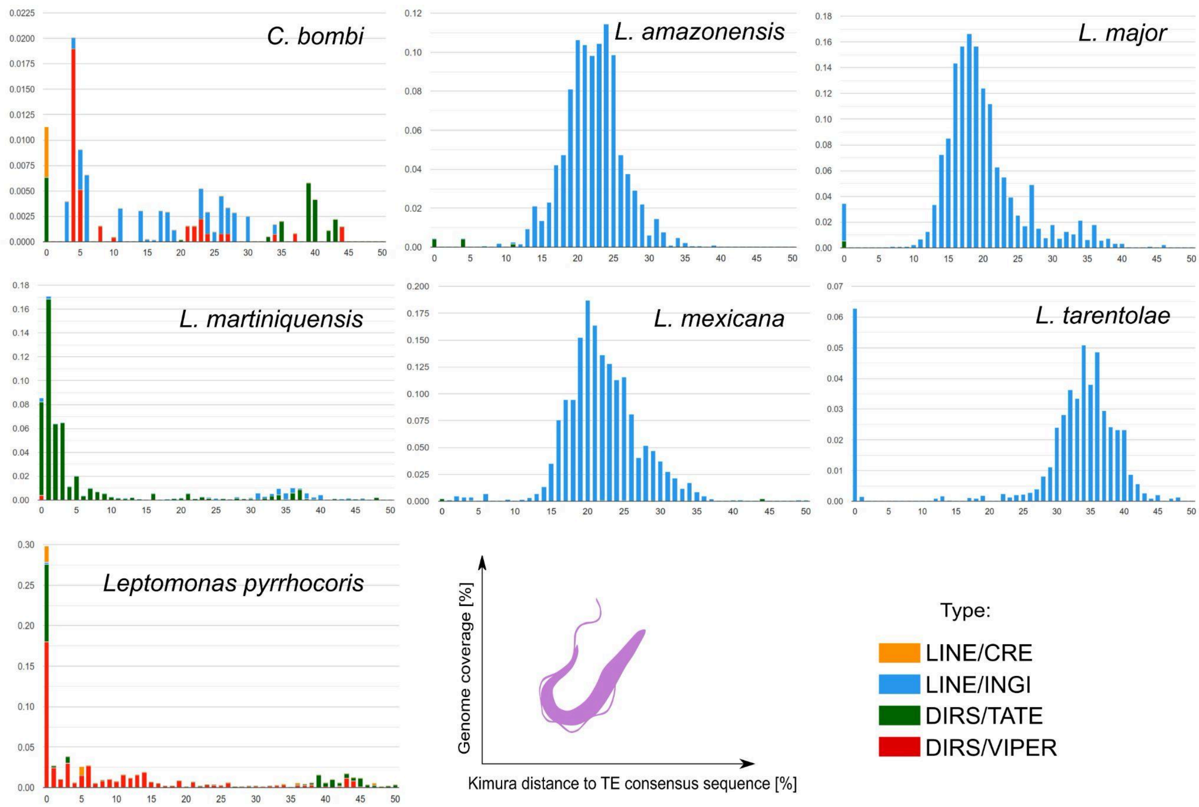

Figure S8. Sequence divergence distribution of TEs. Distribution of sequence divergence of TE families in seven species. The x-axis displays Kimura 2-Parameter sequence divergence between individual TE copies and consensus. The y-axis shows the percentage of the host genomes that is annotated as TEs.
