# Supplementary material for: Comparative analysis of the mobilome yields new insights into its diversity, dynamics and evolution in parasites of the Trypanosomatidae family
Source: Parasitology. 2025 Jun 13;152(6):602–17. doi: 10.1017/S0031182025100231 (PMC12278014; doi:10.1017/S0031182025100231)
Supplement: Tullume-Vergara et al. supplementary material 2 — Tullume-Vergara et al. supplementary material [file S0031182025100231sup002.pdf]

Supplementary Table S1. Source and assembly information on the 57 trypanosomatid genomes used in this study.

Species	Strain	Genome source	Assembly level	Assembly length (bp)	Sequencing coverage	Sequencing technology	Contig N50 (bp)	G+C%	Assembler	Life Cycle	Reference
Angomonas ambiguus	PNG-M02	GCA_019695575.1	Scaffold	23,394,163	31.0x	Illumina	109,297	53.0	Spades v. 3.14.0	Monoxenous	**Skalický et al. 2021**
Angomonas deanei	PRA-265	GCA_903995115.1	Chromosome	20,976,081	665.0x	ONT + Illumina	646,966	49.0	Canu v. 1.8	Monoxenous	**Davey et al. 2021**
Blastocrithidia nonstop	p57	GCA_028554745.1	Contig	24,706,389	240.0x	PacBio + Illumina	441,521	33.0	Spades v. 3.9.0	Monoxenous	**Kachale et al. 2023**
Blechomonas sp.	303E	PRJNA1179471	Contig	22,286,162	39.0x	Illumina	61,737	50.3	Newbler v. 2.9	Monoxenous	Unpublished
Crithidia bombi	GDC	GCA_900240985.1	Scaffold	31,660,431	243.0x	PacBio + 454	124,651	55.8	PBjelly v. 12.9.14	Monoxenous	**Schmid-Hempel et al. 2018**
Crithidia expoeki	GDC	GCA_900240875.1	Contig	34,080,000	62.0x	PacBio + 454	592,188	54.4	HGAP v. 2	Monoxenous	**Schmid-Hempel et al. 2018**
Crithidia fasciculata	Cf-Cl	GCA_000331325.2	Chromosome	41,297,378	130.0x	PacBio	778,443	57.0	Celera v. 8.0	Monoxenous	**Warren et al. 2021**
Endotrypanum monterogeii	LV88	GCA_000333855.2	Chromosome	32,086,870	50.0 x	Illumina + 454	33,059	52.5	Newbler v. 2.0.1	Dixenous	**Warren et al. 2021**
Herpetomonas megaseliae	-	ERR842794 (*)	Contig	N.A.	N.A	Illumina	N.A.	58.0	N.A.	Monoxenous	Wellcome Trust Sanger Inst, 2015
Herpetomonas samuelpessoai	TCC005E	PRJNA1179471	Contig	36,331,503	40.0x	PacBio + Illumina	63,573	56.8	Canu v. 2.2	Monoxenous	Unpublished
Kentomonas sorsogonicus	MF-08	GCA_030347455.1	Scaffold	35,671,915	35.0x	PacBio + Illumina	442,622	56.0	Flye v. 2 + pilon2	Monoxenous	**Zavataro et al., 2024**
Lafontella mariadeanei	TCC004E	PRJNA1179471	Contig	37,602,289	46.0x	PacBio + Illumina	68,039	50.9	Canu v. 2.2	Monoxenous	Unpublished
Leishmania aethiopica	209-622	GCA_003992445.1	Contig	33,648,436	74.0x	PacBio + Illumina	763,733	60.0	Canu v. 1.6	Dixenous	**Batra et al. 2019**
Leishmania amazonensis	M2269	N.A	Chromosome	32,360,000	51.0x	ONT + Illumina	1,039,401	59.6	Flye v.2	Dixenous	Unpublished
Leishmania arabica	LEM1108	GCA_000410695.2	Chromosome	30,774.332	94.0x	Illumina	52,119	59.2	AllPaths-LG v. 2015	Dixenous	**Warren et al. 2021**
Leishmania braziliensis	M2904	GCA_900537975.1	Chromosome	32,300,000	100.0x	PacBio + Illumina	1,100,000	58.0	HGAP4	Dixenous	**González de la Fuente et al. 2018**
Leishmania chagasi	M6445	GCA_018291365.1	Contig	35,326,942	150.0x	Illumina	8,377	56.9	Spades v. 3.12	Dixenous	**Silveira et al. 2021**
Leishmania donovani	HU3	GCA_900635355.2	Chromosome	33,000,000	100.0x	PacBio + Illumina	1,100,000	59.5	HGAP3	Dixenous	**Camacho et al. 2019**
Leishmania enriettii	LV763	GCA_017916305.1	Chromosome	33,300,000	271.8x	ONT + Illumina	790,000	59.5	Flye v.2.6	Dixenous	**Almutairi et al. 2021**
Leishmania gerbilli	LEM452	GCA_000443025.1	Scaffold	30,822,621	140.0x	Illumina	57,008	59.6	AllPaths-LG v. 2012	Dixenous	Whashington University SM

Leishmania guyanensis	M4147	JBIEOV000000000	Chromosome	32,271,267	167.0x	PacBio + Illumina	1,067,002	57.8	Flye v. 2	Dixenous	Submitted
Leishmania infantum	JPCM5	GCA_900500625.2	Chromosome	32,134,935	370.0x	PacBio + Illumina	1,055,293	59.0	CLC Bio v. 5.0	Dixenous	**Gonzalez de la Fuente et al. 2017**
Leishmania lainsoni	216-64	GCA_003664395.1	Contig	34,200,000	74.0x	PacBio + Illumina	638,900	57.5	Canu v. 1.6	Dixenous	**Lin et al. 2019**
Leishmania macropodum	AM-2004	SRR8186979 (*)	Contig	29,590,000	45.0x	Illumina	27,960	57.0	Spades v. 3.10.1	Dixenous	**Butenko et al. 2019**
Leishmania major	Friedlin	GCA_916722125.1	Chromosome	32,800,000	95.0x	PacBio + Illumina	1,100,000	59.5	HGAP v. 4.0	Dixenous	**Camacho et al. 2021**
Leishmania martiniquensis	LV760	GCA_017916325.1	Chromosome	32,400,000	277.9x	ONT + Illumina	841,800	59.5	Flye assembler v. 2.6	Dixenous	**Almutairi et al. 2021**
Leishmania mexicana	215-49	GCA_003992435.1	Contig	32,100,000	87.0x	PacBio +Illumina	826,000	59.5	Canu v. 1.6	Dixenous	**Batra et al. 2019**
Leishmania naiffi	CL223	ERX180449 (*)	Contig	30,341,095	56.0x	Illumina	5,700	58.0	Velvet v . 1.2.09	Dixenous	**Coughlan et al. 2018**
Leishmania orientalis	LV768	GCA_017916335.1	Chromosome	34,200,000	390.7x	ONT + Illumina	1,100,000	59.0	Flye assembler v. 2.6	Dixenous	**Almutairi et al. 2021**
Leishmania panamensis	Psc-1	GCA_000755165.1	Chromosome	30,000,000	30.0x	Illumina	97,600	57.5	Newbler v. 2.6 + PAGIT	Dixenous	**Llanes et al. 2015**
Leishmania peruviana	LEM1537	GCA_001403695.1	Scaffold	25,000,000	30.0x	Illumina	1,400	55.0	Velvet v. 1.1.10	Dixenous	**Valdivia et al. 2015**
Leishmania shawi	M8408	JBINZM000000000	Chromosome	32,565,480	36.0x	PacBio + Illumina	937,220	57.7	Flye assembler v. 2	Dixenous	Submitted
Leishmania chancei	LV757	GCA_017918215.1	Chromosome	36,000,000	371.2x	ONT + Illumina	961,600	59.5	Flye assembler v. 2.6	Dixenous	**Kwakye-Nuako et al. 2023**
Leishmania procaviensis	LV425	GCA_017918225.1	Chromosome	34,100,000	291.5x	ONT + Illumina	891,000	59.0	Flye assembler v. 2.6	Dixenous	**Almutairi et al. 2023**
Leishmania tarentolae	Parrot-TarII	GCA_009731335.1	Contigs	35,416,496	120.0x	PacBio	663,000	57.4	HGAP v. 3	Dixenous	**Goto et al. 2020**
Leishmania tropica	216-162	GCA_014139745.1	Chromosome	32,700,000	75.0x	PacBio + Illumina	1,100,000	59.0	Flye assembler v. 2.4	Dixenous	**Unoarumhi et al. 2021**
Leishmania turanica	LEM423	GCA_000441995.1	Scaffold	30,846,294	108.0x	Illumina	39,210	59.5	AllPaths-LG v. 2012	Dixenous	Whashington University SM
Leptomonas pyrrhocoris	H10	GCA_001293395.1	Scaffold	30,400,000	37.0x	PacBio	142,800	56.5	CLC Genomics W v. 7	Monoxenous	**Flegontov et al. 2016**
Leptomonas seymouri	30220	GCA_001299535.1	Scaffold	27,600,000	180.0x	Illumina	24,400	55.5	CLC Genomics W v. 7	Monoxenous	**Kraeva et al. 2015**
Lotmaria passim	422	GCA_037349495.1	Chromosome	33,726,020	161.0x	PacBio + Illumina	1,528,037	54.5	Hifiasm v. 0.19.6	Monoxenous	**Markowitz et al. 2025**
Novymonas esmeralda	E262AT	GCA_019188245.1	Scaffold	32,100,000	150.0x	Illumina	197,811	62.7	Spades v. 3.9.0	Monoxenous	**Zakharova et al. 2021**
Paratrypanosoma confusum	CUL13MS	GCA_002921335.1	Scaffold	25,300,000	31.0x	Illumina	7,000	61.5	Newbler v. 2.9	Monoxenous	**Skalický et al. 2017**
Phytomonas francai	TCC064	GCA_001766655.1	Scaffold	17,700,000	200.0x	Illumina	48,200	48.5	AllPaths v. 2015	Dixenous	**Butter et al. 2017**
Porcisia deanei	TCC258	GCA_018683835.1	Scaffold	29,500,000	167.6x	Illumina	20,100	55.0	SPAdes v. 3.13.0	Dixenous	**ATS Albanaz et al. 2021**

Porcisia hertigi	C119	GCA_017918235.1	Chromosome	34,958,538	177.1x	ONT + Illumina	967,170	56.0	Flye assembler v.2.6	Dixenous	**Almutari et al. 2021**
Sergeia sp.	Isolate 2467	PRJNA1179471	Contig	24,824,878	30.0x	Illumina	9,620	57.6	Newbler 2.9	Monoxenous	Unpublished
Trypanosoma brucei	EATRO1125	GCA_019096175.1	Chromosome	64,100,000	30.0x	PacBio + Illumina	249,300	42.5	Canu v. 2.1	Dixenous	**Naguleswaran et al. 2021**
Trypanosoma congolense	Tc1/148	GCA_002287245.1	Contig	41,200,000	70.0x	PacBio	421,700	47.0	HGAP3 v. 2017	Dixenous	**Abbas et al. 2018**
Trypanosoma cruzi	Dm28c	GCA_003177105.1	Contig	53,300,000	76.0x	PacBio	314,600	51.5	HGAP v. 3	Dixenous	**Berna et al. 2018**
Trypanosoma equiperdum	IVM-t1	GCA_003543875.1	Chromosome	26,988,997	500.0x	PacBio + Illumina	859,800	46.0	HGAP v. 3	Dixenous	**Davaasuren et al. 2019**
Trypanosoma evansi	Stib 805	GCA_917563935.1	Chromosome	25,400,000	57.0x	PacBio	2,400,000	46.5	Generic 1.0	Dixenous	Wellcome Sanger Institute
Trypanosoma grayi	ANR4	GCA_000691245.1	Scaffold	20,952,750	~10x	Illumina	16,756	53.0	AllPaths v. 2014	Dixenous	**Kelly et al. 2014**
Trypanosoma melophagium	St. Kilda	GCA_022059095.1	Contig	23,300,000	225.0x	ONT + Illumina	505,900	41.0	Wtdbg v. 05/2021	Dixenous	**Oldrieve et al. 2022**
Trypanosoma theileri	Edinburgh	GCA_002087225.1	Scaffold	25,700,000	100.0x	Illumina	19,100	40.0	AllPaths v. 2014	Dixenous	**Kelly et al., 2017**
Trypanosoma vivax	IL1392	GCA_021307395.1	Contig	67,800,000	92.0x	PacBio + Illumina	261,200	53.5	HGAP v. 3	Dixenous	Centre for Genomic Research
Vickermania ingenoplastis	Colprot021	GCA_010157825.1	Contig	35,300,321	270.0x	ONT + Illumina	376,290	53.7	Canu v. 1.4	Monoxenous	**Opperdoes et al., 2021**
Zelonia costaricensis	TCC169	GCA_034640465.1	Contig	38,807,521	~23x	454	17,448	64.3	Newbler v. 2.9	Monoxenous	**Tullume et al., 2023**

N.A = Not applicable

References

Abbas, AH, Silva Pereira, S, D'Archivio, S, Wickstead, B, Morrison, LJ, Hall, N, Hertz-Fowler, C, Darby, AC, and Jackson AP (2018). The Structure of a Conserved Telomeric Region Associated with Variant Antigen Loci in the Blood Parasite *Trypanosoma congolense*. *Genome Biology Evolution* **10**(9):2458-2473. doi: 10.1093/gbe/evy186.

Albanaz, ATS, Gerasimov, ES, Shaw, JJ, Sádlová, J, Lukeš, J, Volf, P, Opperdoes, FR, Kostygov, AY, Butenko, A and Yurchenko, V (2021). Genome Analysis of *Endotrypanum* and *Porcisia* spp., Closest Phylogenetic Relatives of Leishmania, Highlights the Role of Amastins in Shaping Pathogenicity. *Genes (Basel)* **12**(3), 444.

Almutairi, H, Urbaniak, MD, Bates, MD, Jariyapan, N, Al-Salem, WS, Dillon, RJ, Bates, PA and Gatherer, D (2021). Chromosome-Scale Assembly of the Complete Genome Sequence of *Leishmania* (Mundinia) *martiniquensis*, Isolate LSCM1, Strain LV760. *Microbiology Resource Announcements* **10**(24):e0005821.

Almutairi, H, Urbaniak, MD, Bates, MD, Jariyapan, N, Kwakye-Nuako, G, Thomaz Soccol, V, Al-Salem, WS, Dillon, RJ, Bates, PA and Gatherer, D (2021). Chromosome-scale genome sequencing, assembly and annotation of six genomes from subfamily Leishmaniinae. *Scientific Data* **8**(1), 234.

Almutairi, H, Urbaniak, MD, Bates, MD, Kwakye-Nuako, G, Al-Salem, WS, Dillon, RJ, Bates, PA and Gatherer, D (2023). Chromosome-Scale Assembly of the Complete Genome Sequence of *Leishmania* (Mundinia) *procaviensis* Isolate 253, Strain LV425. *Microbiology Resource Announcements* **12**(4), e0130622.

Batra, D, Lin, W, Narayanan, V, Rowe, LA, Sheth, M, Zheng, Y, Loparev, V and de Almeida, M (2019). Draft Genome Sequences of *Leishmania (Leishmania) amazonensis, Leishmania (Leishmania) mexicana,* and *Leishmania (Leishmania) aethiopica*, Potential Etiological Agents of Diffuse Cutaneous Leishmaniasis. *Microbiology Resource Announcements* **8**(20),e00269-19.

Berná, L, Rodriguez, M, Chiribao, ML, Parodi-Talice, A, Pita, S, Rijo, G, Alvarez-Valin, F and Robello, C (2018). Expanding an expanded genome: long-read sequencing of *Trypanosoma cruzi*. *Microbial Genomics* **4**(5), e000177.

Butenko, A, Kostygov, AY, Sádlová, J, Kleschenko, Y, Bečvář, T, Podešvová, L, Macedo, DH, Žihala, D, Lukeš, J, Bates, PA, Volf, P, Opperdoes, FR and Yurchenko, V (2019). Comparative genomics of *Leishmania* (Mundinia). *BMC Genomics* **20**(1), 726.

Butler, CE, Jaskowska, E and Kelly, S (2017). Genome Sequence of *Phytomonas françai*, a Cassava (Manihot esculenta) Latex Parasite. *Genome Announcements* **5**(2), e01266-16.

Camacho, E, González-de la Fuente, S, Rastrojo, A, Peiró-Pastor, R, Solana, JC, Tabera, L, Gamarro, F, Carrasco-Ramiro, F, Requena, JM and Aguado, B (2019). Complete assembly of the *Leishmania donovani* (HU3 strain) genome and transcriptome annotation. *Scientific Report* **9**(1), 6127.

Camacho, E, González-de la Fuente, S, Solana, JC, Rastrojo, A, Carrasco-Ramiro, F, Requen,a JM, and Aguado, B (2021). Gene Annotation and Transcriptome Delineation on a De Novo Genome Assembly for the Reference *Leishmania major* Friedlin Strain. *Genes (Basel)* **12**(9),1359.

Coughlan, S, Taylor, AS, Feane, E, Sanders, M, Schonian, G, Cotton, JA and Downing, T (2018). *Leishmania naiffi* and *Leishmania guyanensis* reference genomes highlight genome structure and gene evolution in the *Viannia* subgenus. *Royal Society Open Science* **5**(4), 172212.

Davaasuren, B, Yamagishi, J, Mizushima, D, Narantsatsral, S, Otgonsuren, D, Myagmarsuren, P, Battsetseg, B, Battur, B, Inoue, N and Suganuma, K (2019). Draft Genome Sequence of *Trypanosoma equiperdum* Strain IVM-t1. *Microbiology Resource Announcuments* **8**(9), e01119-18.

Davey, JW, Catta-Preta, CMC, James, S, Forrester, S, Motta, MCM, Ashton, PD and Mottram, JC (2021). Chromosomal assembly of the nuclear genome of the endosymbiont-bearing trypanosomatid *Angomonas deanei*. *G3 (Bethesda)* **11**(1), jkaa018.

Flegontov, P, Butenko, A, Firsov, S, Kraeva, N, Eliáš, M, Field, MC, Filatov, D, Flegontova, O, Gerasimov, ES, Hlaváčová, J, Ishemgulova, A, Jackson, AP, Kelly, S, Kostygov, AY, Logacheva, MD, Maslov, DA, Opperdoes, FR, O'Reilly, A, Sádlová, J, Ševčíková, T, Venkatesh, D, Vlček, Č, Volf, P, Votýpka, J, Záhonová, K, Yurchenko, V and Lukeš, J (2016). Genome of *Leptomonas pyrrhocoris*: a high-quality reference for monoxenous trypanosomatids and new insights into evolution of *Leishmania*. *Scientific Report* **6**, 23704.

González-de la Fuente, S, Peiró-Pastor, R, Rastrojo, A, Moreno, J, Carrasco-Ramiro, F, Requena, JM, and Aguado, B (2017). Resequencing of the *Leishmania infantum* (strain JPCM5) genome and de novo assembly into 36 contigs. *Scientific Report* **7**(1), 18050.

González-de la Fuente, S, Camacho, E, Peiró-Pastor, R, Rastrojo, A, Carrasco-Ramiro, F, Aguado, B, and Requena, JM (2018). Complete and de novo assembly of the *Leishmania braziliensis* (M2904) genome. *Memorias do Instituto Oswaldo Cruz* **114**, e180438.

Goto, Y, Kuroki, A, Suzuki, K and Yamagishi, J (2020). Draft Genome Sequence of *Leishmania tarentolae* Parrot Tar II, Obtained by Single-Molecule Real-Time Sequencing. *Microbiology Resource Announcements* **9**(21), e00050-20.

Kachale, A, Pavlíková, Z, Nenarokova, A, Roithová, A, Durante, IM, Miletínová, P, Záhonová, K, Nenarokov, S, Votýpka, J, Horáková, E, Ross, RL, Yurchenko, V, Beznosková, P, Paris, Z, Valášek, LS, and Lukeš, J (2023). Short tRNA anticodon stem and mutant eRF1 allow stop codon reassignment. *Nature* **613** (7945), 751-758.

Kelly, S, Ivens, A, Manna, PT, Gibson, W and Field, MC (2014). A draft genome for the African crocodilian trypanosome *Trypanosoma grayi*. *Scientific Data* **1**, 140024.

Kelly, S, Ivens, A, Mott, GA, O'Neill, E, Emms, D, Macleod, O, Voorheis, P, Tyler, K, Clark, M, Matthews, J, Matthews, K and Carrington, M (2017). An Alternative Strategy for Trypanosome Survival in the Mammalian Bloodstream Revealed through Genome and Transcriptome Analysis of the Ubiquitous Bovine Parasite *Trypanosoma* (Megatrypanum) *theileri*. *Genome Biology Evolution* **9**(8),2093-2109.

Kraeva, N, Butenko, A, Hlaváčová, J, Kostygov, A, Myškova, J, Grybchuk, D, Leštinová, T, Votýpka, J, Volf, P, Opperdoes, F, Flegontov, P, Lukeš, J and Yurchenko, V (2015). *Leptomonas seymouri*: Adaptations to the Dixenous Life Cycle Analyzed by Genome Sequencing, Transcriptome Profiling and Co-infection with *Leishmania donovani*. *PLoS Pathogens* **11**(8),e1005127.

Kwakye-Nuako, G, Mosore, MT, Boakye, D and Bates, PA (2023). Description, biology, and medical significance of *Leishmania (Mundinia) chancei* n. sp. (Kinetoplastea: Trypanosomatidae) from Ghana and Leishmania (Mundinia) procaviensis n. sp. (Kinetoplastea: Trypanosomatidae) from Namibia. *The Journal of Parasitology* **109** (1), 43-50.

Lin, W, Batra, D, Narayanan, V, Rowe, LA, Sheth, M, Zheng, Y, Juieng, P, Loparev, V and de Almeida, M (2019). First Draft Genome Sequence of *Leishmania (Viannia) lainsoni* Strain 216-34, Isolated from a Peruvian Clinical Case. *Microbiology Resource Announcements* **8**(6), e01524-18.

Llanes, A, Restrepo, CM, Del Vecchio, G, Anguizola, FJ and Lleonart, R (2015). The genome of *Leishmania panamensis*: insights into genomics of the L. (Viannia) subgenus. *Scientific Report* **5**,8550.

Naguleswaran, A, Fernandes, P, Bevkal, S, Rehmann, R, Nicholson, P and Roditi, I (2021). Developmental changes and metabolic reprogramming during establishment of infection and progression of *Trypanosoma brucei brucei* through its insect host. *PLoS Neglected Tropical Diseases* **15**(9), e0009504.

Oldrieve, GR, Malacart, B, López-Vidal, J and Matthews, KR (2022). The genomic basis of host and vector specificity in non-pathogenic trypanosomatids. *Biology Open* **11**(4), bio059237.

Opperdoes, FR, Butenko A, Zakharova, A, Gerasimov, ES, Zimmer, SL, Lukeš, J and Yurchenko, V (2021). The Remarkable Metabolism of *Vickermania ingenoplastis*: Genomic Predictions. *Pathogens* **10**(1), 68.

Markowitz, L, Nearman, A, Zhao, Z, Boncristiani, D, Butenko, A, de Pablos, LM, Marin, A, Xu, G, Machado, CA, Schwarz, RS, Palmer-Young, EC, and Evans, JD (2024). Somy evolution in the honey bee infecting trypanosomatid parasite *Lotmaria passim*. *G3 (Bethesda)* **15**(1):jkae258. doi: 10.1093/g3journal/jkae258.

Schmid-Hempel, P, Aebi, M, Barribeau, S, Kitajima, T, du Plessis, L, Schmid-Hempel, R and Zoller, S (2018). The genomes of *Crithidia bombi* and *C. expoeki*, common parasites of bumblebees. *PLoS One* **13**(1), e0189738.

Silveira, FT, Sousa-Junior, EC, Silvestre, RV, Costa-Martins, AG, da Costa -Pinheiro, K, Sosa, Ochoa W, Vasconcelos Dos Santos, T, Ramos, PK, Casseb, S, da Silva, SP, Valeriano, CZ, Lima, LV, Campos, MB, da Matta, VL, Gomes, CM, Flores, GV, Pacheco, CM, Corbett, CE, Nakaya, H and Laurenti, MD (2021). Whole-Genome Sequencing of *Leishmania infantum chagasi* Isolates from Honduras and Brazil. *Microbiology Resource Announcements* **10**(48), e0047121.

Skalický, T, Dobáková, E, Wheeler, RJ, Tesařová, M, Flegontov, P, Jirsová, D, Votýpka, J, Yurchenko, V, Ayala, FJ and Lukeš, J (2017). Extensive flagellar remodeling during the complex life cycle of *Paratrypanosoma*, an early-branching trypanosomatid. *Proceedings of the National Academic of Sciences of the United State American* **114**(44),11757-11762.

Skalický, T, Alves, JMP, Morais, AC, Režnarová, J, Butenko, A, Lukeš, J, Serrano, MG, Buck, GA, Teixeira, MMG, Camargo, EP, Sanders, M, Cotton, JA, Yurchenko, V and Kostygov, AY (2021). Endosymbiont Capture, a Repeated Process of Endosymbiont Transfer with Replacement in Trypanosomatids *Angomonas* spp. *Pathogens* **10**(6),702.

Tullume-Vergara, PO, Caicedo, KYO, Tantalean, JFC, Serrano, MG, Buck, GA, Teixeira, MMG, Shaw, JJ and Alves, JMP (2023). Genomes of *Endotrypanum monterogeii* from Panama and *Zelonia costaricensis* from Brazil: Expansion of Multigene Families in Leishmaniinae Parasites That Are Close Relatives of Leishmania spp. *Pathogens* **12** (12), 1409.

Unoarumhi, Y, Batra, D, Sheth, M, Narayanan, V, Lin, W, Zheng, Y, Rowe, LA, Pohl, J and de Almeida, M (2021). Chromosome-Level Genome Sequence of *Leishmania (Leishmania) tropica* Strain CDC216-162, Isolated from an Afghanistan Clinical Case. *Microbiology Resource Announcements* **10**(20), e00842-20.

Valdivia, HO, Reis-Cunha, JL, Rodrigues-Luiz, GF, Baptista, RP, Baldeviano, GC, Gerbasi, RV, Dobson, DE, Pratlong, F, Bastien, P, Lescano, AG, Beverley SM, and Bartholomeu, DC (2015). Comparative genomic analysis of *Leishmania (Viannia) peruviana* and *Leishmania (Viannia) braziliensis*. *BMC Genomics* **16**(1):715. doi: 10.1186/s12864-015-1928-z.

Warren, WC, Akopyants, NS, Dobson, DE, Hertz-Fowler, C, Lye, LF, Myler, PJ, Ramasamy, G, Shanmugasundram, A, Silva-Franco, F, Steinbiss, S, Tomlinson, C, Wilson, RK and Beverley, SM (2021). Genome Assemblies across the Diverse Evolutionary Spectrum of *Leishmania* Protozoan Parasites. *Microbiology Resource Announcements* **10**(35), e0054521.

Zakharova, A, Saura, A, Butenko, A, Podešvová, L, Warmusová, S, Kostygov, AY, Nenarokova, A, Lukeš, J, Opperdoes, FR and Yurchenko, V (2021). A New Model Trypanosomatid, *Novymonas esmeraldas*: Genomic Perception of Its "*Candidatus* Pandoraea novymonadis" Endosymbiont. *mBio*. **12** (4),e0160621.

Zavataro, ALE, Skýpalová, K, Vergara, POT, Silva, FM, Butenko, A, Yurchenko, V, Kostygov, AY and Alves, JMP (2024). The genome of the endosymbiont-harboring trypanosomatid *Kentomonas sorsogonicus*. *Protistology* **18** (1), 72-81.
